# Combinational Inhibition of the eIF4F Complex, AKT1, and EZH2 Enhances Anticancer Effects in BRAF^V600E^ Mutant A375 Melanoma Cells

**DOI:** 10.32604/or.2025.071034

**Published:** 2026-02-24

**Authors:** Yuanxin Miao, Fengyun Hao, Sae Hwi Ki

**Affiliations:** 1 Department of Plastic and Reconstructive Surgery, Inha University School of Medicine, Incheon, 22332, Republic of Korea; 2 Department of Plastic Surgery, The Affiliated Hospital of Qingdao University, Qingdao, 266003, China; 3 Department of Pathology, The Affiliated Hospital of Qingdao University, Qingdao, 266003, China; 4 Department of Plastic and Reconstructive Surgery, Inha University Hospital, Incheon, 22332, Republic of Korea

**Keywords:** Melanoma, B-raf proto-oncogene serine/threonine kinase-inhibitor, eukaryotic initiation factor 4F complex inhibitor, extracellular signal-regulated kinases 1/2, enhancer of zeste homolog 2, AKT serine/threonine kinase 1, Bcl-2 modifying factor

## Abstract

**Objectives:**

The eukaryotic initiation factor 4F (eIF4F) translation initiation complex inhibitors (eIF4Fi) were recently found to hyperactivate extracellular signal-regulated kinases 1/2 (ERK1/2) signals, which contribute to acquired resistance to BRAF (B-Raf proto-oncogene, serine/threonine kinase) inhibitors in melanoma. This present study aims to elucidate how to overcome the resistance of the eIF4Fi in BRAF^V600E^ mutant melanoma cells and explore the underlying mechanisms.

**Methods:**

Melanoma A375 (vemurafenib [VEM]-sensitive) and A375R (VEM-resistant) cells were exposed to eIF4Fi RocA at varying doses and durations *in vitro*. We investigated the impact of RocA on the activity of ERK1/2, AKT serine/threonine kinase 1 (AKT1), eIF4E, and enhancer of zeste homolog 2 (EZH2). We then examined the impact of RocA on pro-apoptotic BH3-only proteins and proliferative proteins. We subsequently determined the effect of combined eIF4Fi, AKT1 inhibitor, EZH2 inhibitor or VEM on tumor growth *in vitro* and *in vivo*.

**Results:**

RocA inhibited proliferation and induced apoptosis in A375 cells, but inhibited proliferation in A375R cells. RocA rapidly reactivated ERK1/2 at 3 h and returned to baseline levels at 48 h. However, eIF4E and AKT1 activation began at 12 h and peaked at 48 h. ERK1/2 positively regulated EZH2 and EZH2-dependent expression of c-Fos and EGR1, while AKT1 negatively regulated c-Myc, c-Jun, and BMF, but positively regulated eIF4E. RocA downregulated ERK1/2 (or EZH2, AKT1, and eIF4E) independent bcl-2 and Mcl-1 expression. AKT1i enhanced RocA-induced cell apoptosis, while EZH2i reduced RocA-induced cell proliferation. Combined CR-1-31-B, EZH2i, and AKT1i effectively overcame resistance to RocA and VEM resistance both *in vitro* and *in vivo*.

**Conclusion:**

The eIF4F complex inhibitor reactivates ERK1/2-EZH2 and AKT1 signaling pathways, resulting in resistance to both eIF4Fi and VEM. Combined administration of an eIF4Fi with EZH2 and AKT1 inhibitors effectively enhances sensitivity to both eIF4F complex and BRAF inhibitors.

## 1 Introduction

The B-raf proto-oncogene, serine/threonine kinase (BRAF), represents the most frequently mutated protein kinase in human cancers, particularly in melanoma, where over 50% of patients exhibit activating BRAF mutations, 90% of which occur at amino acid 600 (V600E mutation) [1]. This constitutive activation of BRAF can overactivate the mitogen-activated protein kinase (MAPK) pathway, promoting melanoma proliferation and survival [2]. Targeting this pathway has demonstrated significant therapeutic potential. The BRAF inhibitor VEM (PLX4720), which inhibits the rapidly accelerated fibrosarcoma–extracellular signal-regulated kinase kinase–extracellular signal-regulated kinase (RAF–MEK–ERK, also known as MAPK) pathway, achieved a confirmed response rate of 48%, with only 0.9% of patients showing a complete response, indicating substantial innate resistance [3]. Moreover, acquired resistance typically develops after a median of 6–7 months of treatment in most patients [4]. Although combined treatment with VEM and MEK inhibitor significantly increases progression-free survival in patients with BRAF^V600^ mutation-positive advanced melanoma, resistance to targeted therapies and immunotherapies ultimately emerges [5,6]. Thus, investigating novel mechanisms of acquired resistance is essential for developing therapies that can elicit long-term, sustained responses.

The eukaryotic initiation factor 4F (eIF4F) is a heterotrimeric complex comprising eIF4E, eIF4A, and eIF4G, and is essential for cap-dependent translation in eukaryotic cells [7]. Proper formation of the eIF4F complex requires adequate levels of each eIF4F component. Abnormal activity of this complex has been observed in many cancers and induces the expression of various irregular proteins that promote tumor cell survival, proliferation [8,9], resistance to cancer treatments [10–12], and metastasis [13,14].

Research indicates that the eIF4F translation initiation complex is associated with melanoma resistance to clinical drugs targeting BRAF and MEK kinases [8,9]. However, the mechanisms underlying melanoma cell resistance to eIF4F complex inhibitors during initial or later treatment stages remain incompletely understood. Boussemart et al. [9] demonstrated that combinations of drugs targeting BRAF to inhibit ERK1/2 and eIF4F may overcome resistance in BRAF^V600^-mutant melanoma. Similarly, Valcikova et al. revealed that targeting eIF4F can promote ERK1/2 hyperactivation in melanomas with BRAF or neuroblastoma RAS viral (v-ras) oncogene homolog (NRAS) mutations [8]. Additionally, reactivation of ERK1/2 has been implicated in acquired resistance to eIF4F complex inhibitors [8,9]. Although combinations of drugs targeting MEK/ERK reactivation and eIF4F inhibition may partially overcome resistance [9], these findings suggest that additional signaling pathways, beyond ERK1/2 activation, may also contribute to the development of acquired resistance to eIF4F complex inhibitors.

Enhancer of zeste homolog 2 (EZH2) functions as a key epigenetic regulator and epithelial–mesenchymal transition (EMT) inducer, contributing to various cancer metastases [13,14], cell proliferation, and apoptosis regulation [15,16]. Human melanomas frequently exhibit EZH2 amplification, which drives metastasis in BRAF- and NRAS-mutant melanomas [14] and regulates melanoma invasion and metastasis through activation of serum response factor (SRF)- and Notch-dependent transcription [17]. Consequently, EZH2 has emerged as a critical therapeutic target in cancer. However, the mechanisms underlying EZH2 upregulation in melanoma remain unclear. Previous studies have demonstrated that the MEK/ERK1/2 pathway can activate EZH2 signals in breast cancer cells [18], while activation of the survival kinase protein kinase B (Akt) regulates methylation activity through EZH2 phosphorylation, contributing to oncogenesis [19]. eIF4E, a translational regulator acting downstream of Akt and mTOR, replicates the role of Akt in tumorigenesis and drug resistance [20]. Based on these findings, we hypothesize that eIF4F inhibition may reactivate ERK and/or AKT signals, leading to upregulation of EZH2 signals and subsequent acquired resistance. Moreover, activated AKT upregulates eIF4E activity, reinforcing resistance mechanisms [20]. Furthermore, both EZH2 inhibitors (EZH2i) and AKT inhibitors (AKTi) inhibit apoptosis through pro-apoptotic BMF inhibition, contributing to drug resistance [20,21]. We propose that synergistic therapeutic effects occur through combined inhibition of the eIF4F complex, EZH2, and AKT pathway, thereby reducing resistance mechanisms that restrict the efficacy of anti-BRAF and anti-MEK cancer therapies. Treatment with an eIF4F translation initiation complex inhibitor has been shown to activate EGR1 and c-Fos while inactivating c-Jun and c-Myc [8]. EGR1 and c-Fos are primarily associated with cell proliferation, while c-Jun and c-Myc are implicated in the regulation of cell apoptosis. However, the precise regulation mechanisms and biological functions of these downstream signaling molecules in eIF4F inhibitor-treated melanoma cells require further investigation.

Inhibition of the eIF4F complex, either by blocking the eIF4E-eIF4G interaction or by targeting eIF4E or eIF4A, inhibits tumor growth and restores sensitivity to various anticancer therapies [8,9,22]. However, the mechanisms by which eIF4F complex inhibition regulates these signals and their biological functions remain unclear. In the present study, we investigated the mechanism of eIF4F complex inhibitors on BRAFV600E mutant A375 melanoma cells and how to reverse resistance to eIF4F complex inhibitors.

## 2 Materials and Methods

### 2.1 Ethical Approval Statement

This study was approved by the Affiliated Hospital of Qingdao University, Qingdao, China (QY202386). All animal experiments were conducted following the guidelines of the institutional animal committee of Qingdao University

### 2.2 Cell Culture

The human melanoma cell line A375 was obtained from the American Type Culture Collection (ATCC) (Manassas, VA, USA). Cells were cultured in Dulbecco’s Modified Eagle Medium (DMEM) (Thermo Fisher Scientific, Waltham, MA, USA) supplemented with 10% fetal bovine serum (Thermo Fisher Scientific) and 1% penicillin–streptomycin (Sigma Aldrich, St. Louis, MO, USA). Cultures were maintained at 37°C in a humidified atmosphere containing 5% CO_2_. All cell lines were verified to be mycoplasma-free before the experiment. The identities of the cell lines were confirmed through short tandem repeat validation analysis performed by Biosynthetic Inc. (Lewisville, TX, USA) before use. Cells were maintained at about 50%–60% confluence, and the culture media were changed twice a week. Once thawed, the cells were used for a maximum of 4 months in continuous culture to ensure genomic stability and reproducibility.

VEM-resistant cell lines (A375R) were generated by continuous exposure of parental A375 cells to increasing concentrations of VEM (Selleck Chemicals, Houston, TX, USA), from 0.05 to 10 µM over 6 months. The resulting A375R cells were maintained in 2 µM VEM. Before sensitivity assays, A375R cells were cultured in VEM-free media for 10–14 days to eliminate residual drug effects.

### 2.3 Reagents

Rocaglates (RocA), selective inhibitors of the eIF4F complex, dissolved in DMSO at a concentration of 100 mM, were purchased from MedChemExpress (Monmouth Junction, NJ, USA). RocA (Cat No. HY-19356, purity: 96.43%) was dissolved in DMSO to a stock concentration of 100 µM, and CR-1-31-B (Cat. No. HY-136453) was dissolved in DMSO at a concentration of 10 μM. The EZH2 inhibitor tazemetostat was purchased from ApexBio Technology (Houston, TX, USA). The AKT inhibitor MK-2206 and ERK1/2 inhibitor U0126, as well as VEM, were purchased from Selleck Chemicals. Gene-specific siRNAs were purchased from Santa Cruz Biotechnology (Santa Cruz, Dallas, TX, USA), including: eIF4E siRNA (sc-35284), BMF siRNA(sc-45930), c-Myc siRNA(sc-29226), c-Jun siRNA (sc-29223), c-Fos siRNA (sc-44200), and EGR1 siRNA (sc-44203). EZH2 siRNA (#6509) was purchased from Cell Signaling Technology (CST, Danvers, MA, USA). All negative control siRNAs were purchased from Santa Cruz Biotechnology (sc-44231). siRNAs were transfected at a final concentration of 100 nM, and cells were harvested 48 or 72 h after transfection for analysis. Primary antibodies (dilution, 1:1000) against eIF4E (#9742), p-eIF4E (#9741), bcl-2 (#15071), c-Myc (# 9402), Mcl-1 (#4572), c-Jun (#3270), c-Fos (#5348), EGR1 (#4153), t-AKT1 (#2938), p-AKT1 (#9018), p-ERK1/2 (#9101), ERK1/2 (#9102), EZH2 (#4905), BMF (#50542), PUMA (#4976), Bax (#2772), Noxa(#14766), β-actin(#4967),Bad (#9292), and cleaved-caspase-3 (#9661) were purchased from Cell Signaling Technology. Primary antibodies (dilution, 1:1000) against Bim (sc-374358) were purchased from Santa Cruz Biotechnology.

### 2.4 Small Interfering RNA (siRNA) Transfection

For siRNA transfection, A375 and A375R cells were seeded at approximately 40% confluence in OPTI-MEM serum-free medium (Gibco, Waltham, MA, USA) in 6-cm tissue culture plates and transfected with different small interfering RNAs using Lipofectamine RNAiMAX (Invitrogen, Carlsbad, CA, USA). Cells were harvested 48 or 72 h post-transfection. Each transfection experiment was performed at least three times, and the blocking efficiency of the siRNA clones was evaluated by western blotting.

### 2.5 Cell Viability Assay

Cell viability was evaluated using the 3-(4,5-dimethylthiazol-2-yl)-2,5-diphenyl tetrazolium bromide (MTT) assay (Sigma-Aldrich, St. Louis, MO, USA), according to the manufacturer’s instructions. Briefly, A375 and A375R cells were seeded in 96-well plates at a density of 2 × 10^3^ cells per well and allowed to adhere overnight. Cells were then treated with different concentrations (0, 10, 30, 50, 100, 200, and 300 nM) of RocA and cultured in 96-well plates for 72 h or exposed to VEM (1 and 10 μM) in combination with RocA (50, 100, and 200 nM) for 48 or 72 h. Additionally, cells were transfected with different siRNAs or treated with specific inhibitors for 16 h before exposure to different concentrations of RocA for 72 h. Subsequently, the medium was replaced with 100 μL of fresh medium containing 10 µL of 5 mg/mL MTT reagent, and the plates were incubated at 37°C for 4 h. The medium was then aspirated, and formazan crystals were dissolved in isopropanol containing 0.04 N HCl. Absorbance was measured at 570 nm with a reference wavelength of 750 nm using a Varioskan LUX multimode microplate reader (Thermo Fisher Scientific).

To determine the effect of EZH2, c-Fos, and EGR1 on RocA-induced cell viability, A375 cells were transfected with EZH2 siRNA, EGR1 siRNA, or c-Fos siRNA or co-transfected with EGR1 siRNA + c-Fos siRNA for 16 h. Cells were then treated with RocA (50 and 100 nM) for 72 h, and cell viability was assessed by the MTT assay, as described above.

To determine whether pharmacological inhibition of EZH2 and AKT1 influences RocA-induced cell viability, A375 and A375R cells (2 × 10^3^) were exposed to tazemetostat (2.5 and 5 µM) + RocA (50 and 100 nM), MK-2206 (2.5 and 5 µM) + RocA (50 and 100 nM), or tazemetostat (2.5 and 5 µM) + MK-2206 (2.5 and 5 µM) + RocA (50 and 100 nM). Cells were cultured in 96-well plates for 72 h, and cell viability was assessed by the MTT assay, as described above. To determine the effect of EZH2 and AKT1 inhibition on VEM sensitivity, A375 and A375R cells (2 × 10^3^) were exposed to VEM (1 and 10 µM) + tazemetostat (2.5 µM), VEM (1 and 10 µM) + MK-2206 (2.5 µM), or VEM (1 and 10 µM) + tazemetostat (2.5 µM) + MK-2206 (2.5 µM) and cultured in 96-well plates for 72 h. Cell viability was assessed by the MTT assay, as described above.

### 2.6 Terminal Deoxynucleotidyl Transferase dUTP Nick End Labelling (TUNEL) Staining

Apoptotic cell death was assessed using the TUNEL fluorescence assay kit (Cat. No. C1089, Beyotime, Shanghai, China) according to the manufacturer’s protocol. Briefly, A375 and A375R cells were seeded in 6-well plates at a density of 3 × 10^5^ cells/well and treated with the indicated concentrations of RocA for 48 h. In parallel experiments, cells were transfected with the specified siRNAs (100 nM) for 16 h, followed by exposure to various concentrations of RocA for 48 h. At different experimental time points, cells from each experimental group were seeded onto a 24-well plate containing coverslips. Following incubation with the labeling working solution, 10% phosphate-buffered saline (PBS; pH 7.4, P0149B; Beyotime) wash, addition of DAPI working solution (C1006; Beyotime) and anti-fluorescence quenching agent, the slides were sealed and examined under a fluorescence microscope IX73 (Olympus Corporation, Tokyo, Japan) at 200× magnification, with apoptotic nuclei identified by red fluorescence.

### 2.7 Western Blot Assay

For immunoblotting experiments involving RocA treatment *in vitro*, A375 and A375R cells were treated with 100 nM RocA for 1, 3, 6, 12, 18, 24, 36, and 48 h, or with 50, 100, or 200 nM RocA for 48 h. Whole-cell lysates were collected at the indicated time points for protein analysis.

For combination treatment assays, A375 cells were pre-treated with 2.5 μM of MK-2206 for 2 h or 10 μM of U0126 for 4 h, followed by treatment with 100 nM RocA for 1, 3, 6, 12, 18, 24, 36, and 48 h.

For siRNA transfection experiments, siRNAs (siRNA1, siRNA2 and siRNA3) were transfected at a final concentration of 100 nM for analysis (We chose siRNA with the highest transfection efficiency as the research object): ① A375 cells were transfected with EZH2 siRNA (100 nM) for 16 h, followed by 100 nM RocA treatment for 1, 3, 6, 12, 18, 24, 36, and 48 h; ② A375 cells were transfected with EGR1 siRNA (100 nM) or c-Fos siRNA (100 nM) for 16 h, and then treated with RocA (50 and 100 nM) for 72 h; ③ Cells were transfected with c-Myc siRNA, c-Jun siRNA, or BMF siRNA (100 nM each) for 16 h and then co-treated with RocA (50, 100, or 200 nM) and MK-2206 (1.25, 2.5, or 5 μM) for 48 h; ④ Cells were transfected with EIF4EsiRNA for 16 h and then treated with RocA (50, 100, and 200 nM) for 48 h.

After treatment, cells were lysed at the indicated time points using standard radioimmunoprecipitation assay (RIPA) buffer (Cat. No. 89901, Thermo Fisher Scientific) containing 1% protease inhibitors (Cat. No. 36978, Thermo Fisher Scientific) and PhosphoStop phosphatase inhibitor cocktails (Cat. Nos. P5726, Sigma-Aldrich). Lysates were vortexed, sonicated, and centrifuged, and supernatants were stored at −80°C until use. Protein concentrations were determined using the BCA protein assay kit (Cat. No. P0012S Beyotime). All experiments were performed in triplicate (*n* = 3). Equal amounts of proteins (20–40 µg/lane) were separated on a 4%–12% sodium dodecyl sulfate-polyacrylamide gel (SDS-PAGE) under constant voltage of 120 V for approximately 90 min, followed by transfer to nitrocellulose membranes at 100 V for 60 min. Membranes were blocked for 1 h in 5% (w/v) bovine serum albumin (Sigma-Aldrich) and incubated with primary antibodies overnight at 4°C. The primary antibodies included anti-(p)-ERK1/2, anti-(p)-AKT1, anti-EZH2, anti-BMF, anti-c-Myc, anti-c-Jun, anti-c-Fos, anti-EGR1 anti-bcl-2, anti-Mcl-1, anti-PUMA, anti-Bim, anti-Noxa, anti-Bax, anti-Bad, anti-cleaved-caspase-3, and anti-β-actin. The dilution factor for each primary antibody was established based on the manufacturer’s recommendations and validated by preliminary experiments.

Following incubation with primary antibodies, membranes underwent three 10-min washes with TBST buffer solution. They were then incubated with horseradish peroxidase (HRP)-conjugated secondary antibodies (Cat. No. BA1054, Boster, Wuhan, China; 1:10,000) at room temperature for 1 h. Protein bands were visualized using the enhanced chemiluminescence (ECL) detection kit (Cat. No. RPN2209, Amersham International, Amersham, UK), according to the manufacturer’s protocol. Band intensities were quantified using Image J software 3.0 (Bio-Rad Laboratories Inc., Hercules, CA, USA). Original western blot data are reported as WB Original Data. For immunoblots examining combined treatment with VEM + CR-1-31-B (CR-31) + MK-2206 + tazemetostat treatment *in vivo*, tumor tissues from xenografts were homogenized using a Fisher Scientific homogenizer, lysed in RIPA buffer, and 30 µg/lane of protein lysates were separated by SDS-PAGE followed by immunoblotting with the indicated antibodies, as described above.

### 2.8 Animals and Tumor Growth Analysis

All *in vivo* experiments were approved by the Affiliated Hospital of Qingdao University, Qingdao, China. Female BALB/c-nu mice aged 4–6 weeks (*n* = 6 per group) were obtained from Doo Yeol Biotech (SLRC Laboratories, Shanghai, China). A375 and A375R cells (5 × 10^6^) suspended in 200 µL of sterile medium were subcutaneously injected into the flank region of each mouse [23]. For long-term therapeutic response analysis, treatment was initiated 5 days after tumor cell injection. Mice received one of the following regimens: 20 mg/kg VEM i.p for 14 days, 0.2 mg kg^−1^ CR-31/mouse/day i.p for 14 days + 20 mg/kg VEM i.p for 14 days, or a combination of 20 mg/kg VEM i.p for 14 days + 0.2 mg kg^−1^ CR-31/mouse/day i.p for 14 days + MK-2206 (120 mg/kg, orally, three times weekly for 14 days) + tazemetostat (400 mg/kg oral gavage, bis in die (b.i.d.) for 10 days).

In the short-term therapeutic response group, treatments were administered daily for 5 consecutive days, and animals were sacrificed 6 h after the final treatment. Tumors were excised, lysed in RIPA buffer, and analyzed via western blotting. For long-term therapeutic response assessment, tumor size was measured every two days using a digital caliper. Tumor volume was calculated using the equation V = (W^2^ × L)/2, where *W* represents the tumor width and *L* the tumor length.

### 2.9 Statistical Analysis

Statistical analyses were conducted using IBM SPSS Statistics Grad Pack 22.0 BASE DOWNLOAD-Win (IBM, Armonk, NY, USA). Data are presented as the mean ± SEM, with all experiments performed in triplicate. One-way ANOVA followed by Tukey’s post hoc test was used for multiple group comparisons, while two-group comparisons were analyzed using Student’s *t*-test, where appropriate. Statistical significance was defined as *p < 0.05, **p < 0.01, ***p < 0.001, and ****p < 0.0001.

## 3 Results

### 3.1 RocA Inhibits Cell Proliferation and Induces Apoptosis in A375 Cells

**Figure 1:**
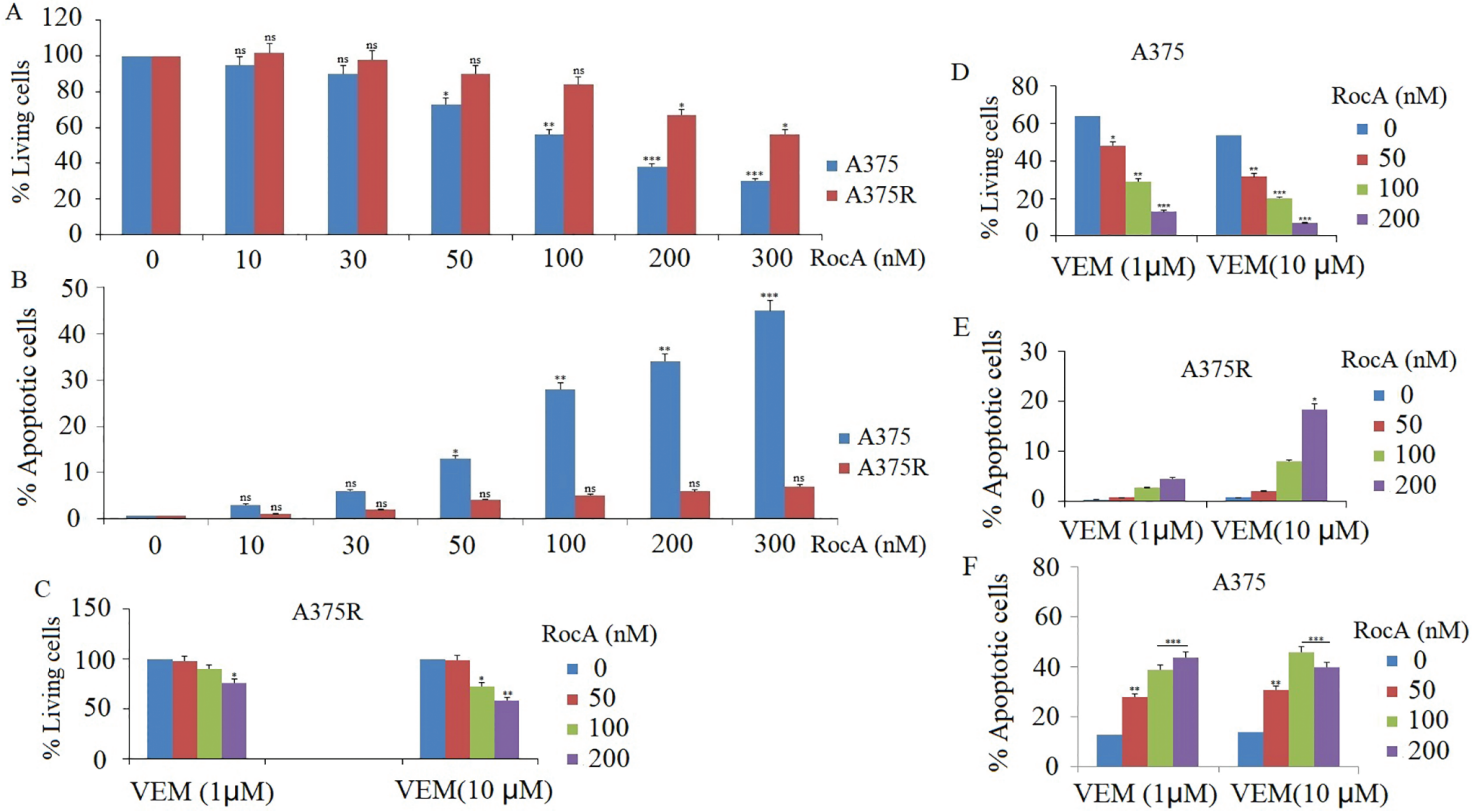
Effect of RocA on cell proliferation and apoptosis in A375 cells. (**A**) A375 and A375R cells were treated with different concentrations of RocA, namely 10, 30, 50, 100, 200, and 300 nM for 72 h. Cell viability was detected by MTT assay. (**B**) A375 and A375R cells were treated with different concentrations of RocA, namely 10, 30, 50, 100, 200, and 300 nM for 48 h. Cell apoptosis was detected by TUNEL assay. (**C**,**D**) A375 and A375R cells were co-treated with VEM (1 and 10 μM) and RocA (50, 100, and 200 nM) for 72 h. Cell viability was assessed by MTT assay. (**E**,**F**) A375 or A375R cells were co-treated with VEM (1 and 10 μM) and RocA (50, 100, and 200 nM) for 72 h. Cell apoptosis was detected by TUNEL assay. Values represent the mean ± SEM. Statistically significant differences are reported in the graph as *p*-values (Student’s *t*-test). ns, not significant, *p < 0.05, **p < 0.01, ***p < 0.001

The eIF4F complex inhibitor RocA functions by inhibiting the eIF4A helicase subunit of the eIF4F complex and enhancing eIF4A RNA affinity, resulting in loss of eIF4A helicase activity [24]. Previous research demonstrates that RocA effectively inhibits cancer cell growth *in vitro* and tumor development *in vivo* [22]. This study initially examined the effect of RocA on cell viability in VEM-sensitive melanoma cells (A375) and VEM-resistant melanoma cells (A375 R). Cells were exposed to varying RocA concentrations (10, 30, 50, 100, 200, and 300 nM) for 72 h. Cell viability assessment via MTT assay revealed significant dose-dependent decreases in both cell lines, with A375 cells showing greater sensitivity to RocA treatment (Fig. 1A).

Following 48-h treatment with RocA at concentrations of 10, 30, 50, 100, 200, and 300 nM, apoptosis was evaluated using the TUNEL assay. The results demonstrated that RocA treatment induced a significant dose-dependent apoptosis in A375 cells (Fig. 1B and Fig S1). In contrast, A375R cells showed minimal apoptotic response to RocA treatment (Fig. 1B).

The study subsequently investigated whether RocA enhanced the sensitivity of A375 and A375R cells to VEM. Both cell lines were treated with VEM (1 and 10 μM) and RocA (50, 100, and 200 nM) for 48 h (for apoptosis detection) and 72 h (for cell viability assessment). The combination treatment significantly increased cell apoptosis (Fig. 1E,F) (Figs. S2 and S3) and reduced cell viability (Fig. 1C,D) in both cell types. Notably, the VEM-sensitive A375 cells exhibited a greater enhancement in drug sensitivity following RocA co-treatment compared with the resistant A375R cells.

### 3.2 RocA Treatment Hyperactivates ERK1/2 and Regulates EZH1-Dependent EGR1 and c-Fos Expression

**Figure 2:**
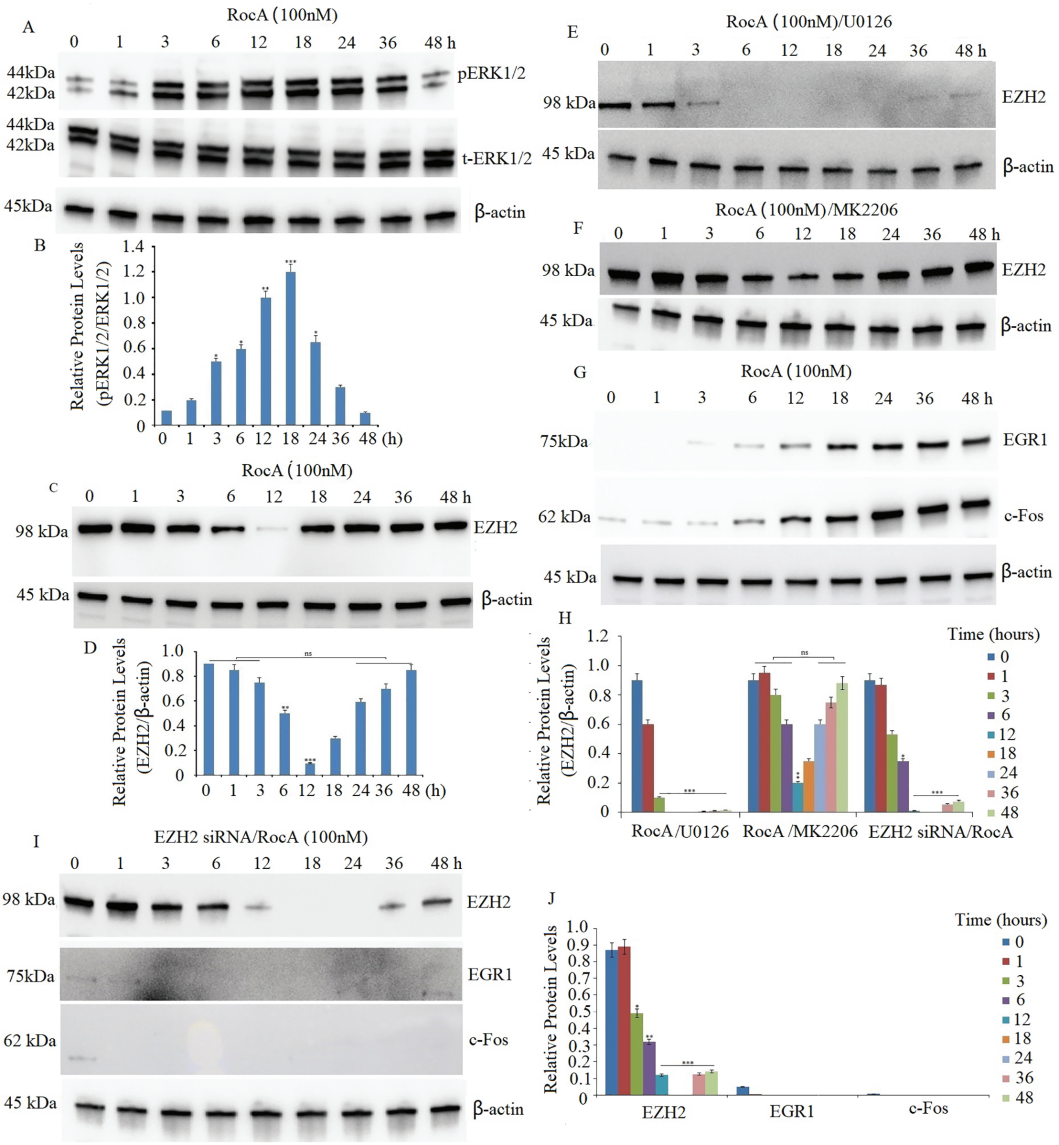
eIF4F inhibition promotes hyperactivation of the ERK/EZH2 pathway, leading to EZH2-driven upregulation of EGR1 and c-Fos. (**A**) A375 cells were treated with 100 nM RocA for 1, 3, 6, 12, 18, 24, 36, and 48 h. Western blot analysis evaluated p-ERK1/2 and ERK1/2 protein expression in RocA-treated cells. The blot was reprobed with β-actin antibody to verify equal protein loading. (**B**) Results represent the mean ± SEM of two independent experiments. *p < 0.05, **p < 0.01, ***p < 0.001. (**C**) A375 cells were treated with 100 nM RocA for 1, 3, 6, 12, 18, 24, 36, and 48 h. Western blot analysis evaluated EZH2 protein expression in RocA-treated cells. The blot was reprobed with β-actin antibody to verify equal protein loading. (**D**) Results represent the mean ± SEM of two independent experiments. ns, not significant, **p < 0.01, ***p < 0.001. A375 cells were exposed to U0126 (10 µM) for 4 h (**E**) or MK2206 (2.5 µM) for 2 h (**F**), followed by treatment with 100 nM RocA for 1, 3, 6, 12, 18, 24, 36, and 48 h. EZH2 protein expression was detected by western blotting. (**G**) A375 cells were exposed to 100 nM RocA for 1, 3, 6, 12, 18, 24, 36, and 48 h. EZH2 protein expression was detected by western blotting. (**H**) Results of EZH2 protein expression represent the mean ± SEM of two independent experiments. *p < 0.05, **p < 0.01, ***p < 0.001, ns, no significance. (**I**) A375 cells were transfected with EZH2 siRNA for 16 h and then treated with 100 nM RocA for 1, 3, 6, 12, 18, 24, 36, and 48 h. The EZH2, c-Fos, and EGR1 protein expressions were detected by western blotting. (**J**) Results of the EZH2, c-Fos, and EGR1 protein expression represent the mean ± SEM of triplicate independent experiments. *p < 0.05, **p < 0.001, ***p < 0.001

Treatment of A375 cells with 30–200 nM RocA for 20 h induced ERK1/2 hyperactivation in a dose-dependent manner [13]. In the present study, A375 cells were treated with 100 nM RocA for 1, 3, 6, 12, 18, 24, 36, and 48 h. The levels of p-ERK1/2 increased progressively from 1 h after RocA administration, peaked at 18–24 h, and subsequently decreased to baseline levels by 48 h (Fig. 2A,B).

EZH2 plays a crucial role in regulating proliferation in basal epidermal cells, with reduced proliferative capacity observed in cells lacking EZH2 [25]. Previous research demonstrated that EIF4E inhibition suppressed EZH2 and CDK1 protein expression, resulting in decreased proliferation of human epidermal keratinocytes [26]. To investigate whether eIF4F regulated EZH2 expression, we first confirmed abundant EZH2 expression in A375 cells through western blot analysis. Treatment of A375 cells with 100 nM RocA led to a significant reduction in EZH2 levels after 12 h, followed by a gradual increase and sustained high expression at 48 h (Fig. 2C,D). Previous studies have shown that ERK1/2 signaling positively regulates EZH2 expression, correlating with aggressive breast cancer subtypes [18]. Additionally, MEK-ERK and PI3K/AKT signaling modulate EZH2 expression in KRAS-mutant colon and pancreatic cancer cell lines [27]. To explore this regulatory mechanism in melanoma, A375 cells were pretreated with U0126 (10 µM) for 4 h or AKT inhibitor MK-2206 (2.5 µM) for 2 h, followed by treatment with 100 nM RocA for various time intervals. U0126 treatment completely suppressed RocA-induced EZH2 upregulation (Fig. 2E,H), while MK-2206 pretreatment did not affect EZH2 reactivation (Fig. 2F,H), indicating that targeting hyperactive ERK1/2 inhibited RocA-induced EZH2 reactivation.

Previous research has shown that eIF4F inhibitor treatment elevated EGR1 and c-Fos levels, correlating with increased ERK activity in BRAFV600E and NRAS-mutant melanoma cell lines [13]. To determine whether EGR1 and c-Fos expression are regulated by EZH2, A375 cells were treated with 100 nM RocA for various time intervals. EGR1 and c-Fos protein levels increased gradually, reaching maximum levels at 48 h (Fig. 2G,H). However, when A375 cells were co-treated with EZH2 siRNA (100 nM) and 100 nM RocA, the reduction in EZH2 expression led to complete inhibition of RocA-induced EGR1 and c-Fos expression (Fig. 2I,J), demonstrating EZH2-dependent regulation of these proteins.

### 3.3 Targeting EZH2 Enhances RocA Sensitivity by Inhibiting Cell Viability, but Does Not Affect Cell Apoptosis

**Figure 3:**
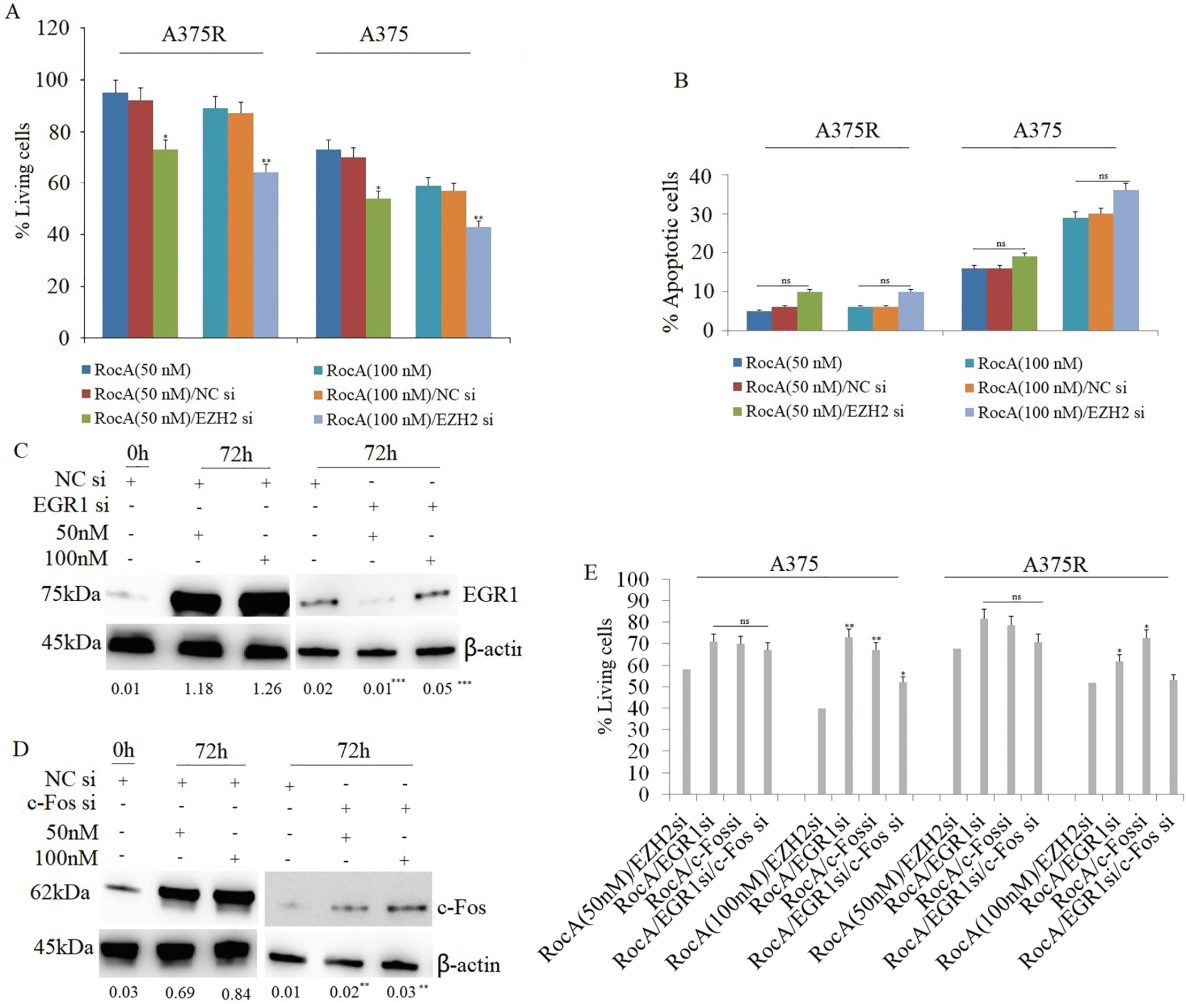
Targeting EZH2 enhances the sensitivity of RocA to A375 cells by inhibiting EGR1 and c-Fos. (**A**) A375 cells were transfected with EZH2 siRNA or NC siRNA for 16 h, followed by RocA treatment (50 and 100 nM) for 72 h. Cell viability was assessed using the MTT assay. (**B**) A375 cells were transfected with EZH2 siRNA or NC siRNA for 16 h, followed by RocA treatment (50 and 100 nM) for 48 h. Cell apoptosis was evaluated using the TUNEL assay. (**C**) A375 cells were transfected with NC siRNA or EGR1 siRNA for 16 h, followed by RocA treatment (50 and 100 nM) for 72 h. EGR1 expression was analyzed by western blotting. (**D**) A375 cells were transfected with NC siRNA or c-Fos siRNA for 16 h, followed by RocA treatment (50 and 100 nM) for 72 h. c-Fos expression was analyzed by western blotting. (**E**) A375 cells were transfected with EZH2 siRNA, EGR1 siRNA, c-Fos siRNA, or co-transfected with EGR1 siRNA + c-Fos siRNA for 16 h, followed by RocA treatment (50 and 100 nM) for 72 h. Cell viability was assessed using the MTT assay. Data are presented as the mean ± SEM. ns, not significant, *p < 0.05; **p < 0.01; ***p < 0.001

The study examined whether targeting EZH2 enhances RocA sensitivity in A375 cells. A375 and A375R cells were transfected with EZH2 siRNA (100 nM) for 16 h, followed by RocA treatment (50 and 100 nM) for 48 or 72 h. MTT assay demonstrated that the combined treatment significantly inhibited cell viability in both cell lines (Fig. 3A). To further determine whether this combination affected cell apoptosis, A375 cells were treated under the same conditions and analyzed for apoptotic activity. The results indicated that co-treatment with EZH2 siRNA and RocA did not significantly enhance cell apoptosis compared to either treatment alone (Fig. 3B and Fig. S4), indicating that EZH2 targeting primarily enhances RocA sensitivity by inhibiting cell proliferation rather than inducing apoptosis.

Subsequently, the study investigated whether targeting EZH2 sensitized A375 cells to RocA through EGR1 or c-Fos. A375 cells were transfected with EGR1 siRNA or c-Fos siRNA for 16 h, followed by RocA treatment (50 and 100 nM) for 72 h. Western blot analysis confirmed that EGR1 (Fig. 3C) and c-Fos (Fig. 3D) expression decreased significantly following their respective siRNA transfections. Cell viability analysis revealed that silencing EGR1 or c-Fos partially restored cell viability following RocA treatment (Fig. 3E). The combination of RocA treatment with either EGR1 siRNA or c-Fos siRNA significantly increased cell viability, although complete recovery was not achieved (Fig. 3E). These findings suggest that EZH2 influences A375 cell sensitivity to RocA, at least in part, through regulation of EGR1 and c-Fos expression. Similarly, in A375R cells, EZH2 silencing sensitized cells to RocA treatment, and co-transfection with EGR1 or c-Fos siRNA led to a comparable partial restoration of cell viability without complete recovery (Fig. 3E).

### 3.4 RocA Treatment Activates AKT1 and Inhibits c-Myc, c-Jun, and BMF

**Figure 4:**
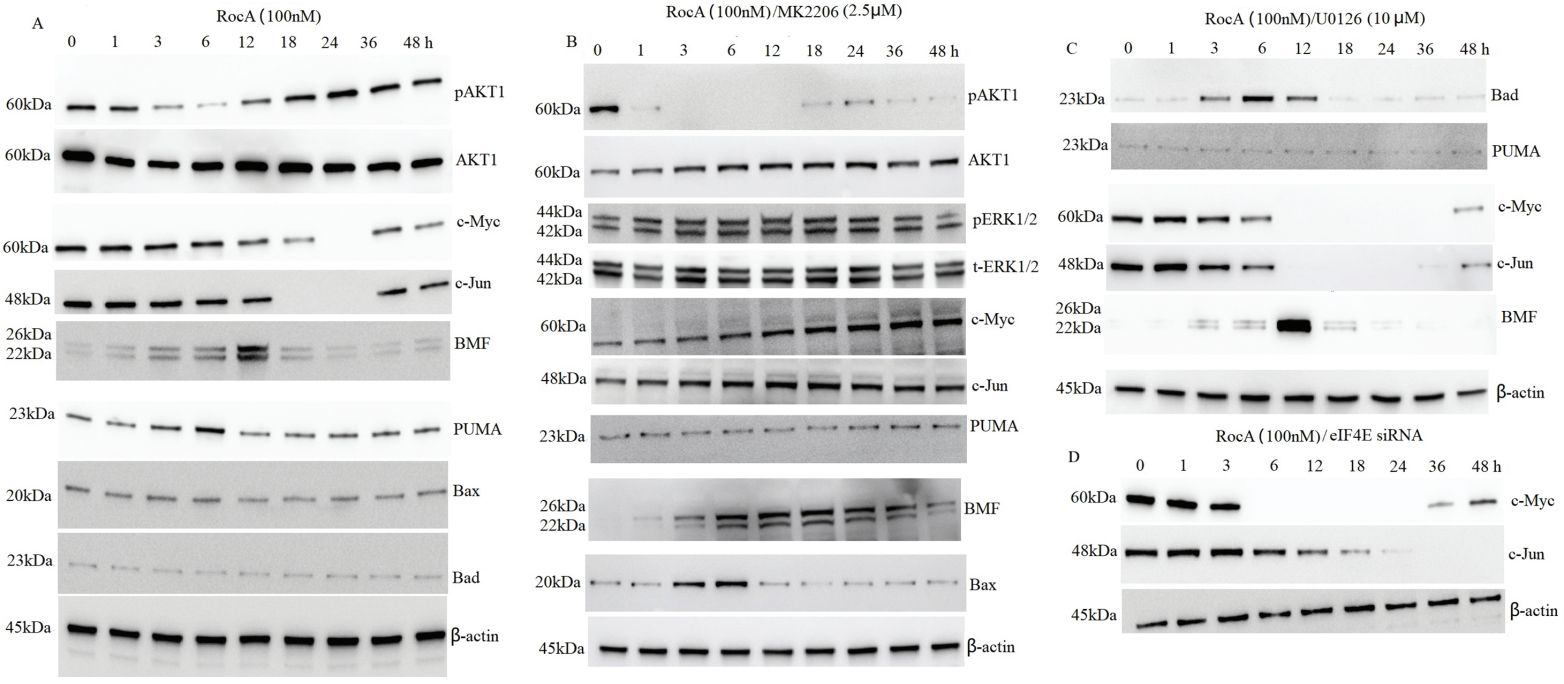
RocA promotes the reactivation of AKT1, leading to AKT1-driven downregulation of c-Myc, c-Jun, and the pro-apoptotic BH3-only protein BMF. A375 cells were treated with 100 nM RocA for 1, 3, 6, 12, 18, 24, 36, and 48 h. (**A**). Alternatively, cells were co-treated with 100 nM RocA and 2.5 μM MK-2206, (**B**) U0126, (**C**) or eIF4E siRNA (**D**) for the same time. The protein levels of AKT1 and its downstream targets were analyzed by western blotting

Treatment with eIF4F inhibitors has been shown to reduce c-Myc and c-Jun expression, while treatment with the specific MEK inhibitor PD184352 failed to increase pro-apoptotic c-Myc or c-Jun expression [13], indicating that these transcription factors are not regulated by ERK1/2 signaling. Previous research has demonstrated that AKT1 regulates eIF4F complex formation and modulates mRNA translation, resulting in activation of a caspase-dependent apoptotic cascade and growth inhibition [14]. To determine whether AKT1 regulates c-Myc and c-Jun, A375 cells were treated with 100 nM RocA for 1, 3, 6, 12, 18, 24, 36, and 48 h. Western blot analysis revealed that p-AKT1 levels decreased in a time-dependent manner after 6 h of RocA treatment, followed by a gradual increase and peak activation at 24 h, before returning to baseline by 48 h (Fig. 4A).

A375 cells were exposed to 2.5 μM AKT inhibitor MK-2206 for 2 h, followed by treatment with 100 nM RocA for 1, 3, 6, 12, 18, 24, 36, and 48 h. MK-2206 abolished RocA-induced AKT1 reactivation in a time-dependent manner (Fig. 4B). However, MK-2206 did not affect RocA-induced ERK1/2 activation (Fig. 4B) compared to RocA treatment alone (Fig. 2A), suggesting that RocA-induced ERK1/2 reactivation occurs through an AKT1-independent pathway. To further investigate whether AKT1 regulates c-Myc and c-Jun, A375 cells were exposed to 2.5 μM MK-2206 for 2 h, followed by 100 nM RocA treatment for various time points. As shown in Fig. 4B, untreated A375 cells exhibited high basal levels of c-Myc and c-Jun. RocA treatment reactivated p-AKT1 and suppressed c-Myc and c-Jun expression, whereas MK-2206 treatment inhibited p-AKT1 and restored c-Myc and c-Jun expression. In contrast, U0126 treatment did not affect RocA-induced c-Myc and c-Jun downregulation (Fig. 4C), compared to RocA treatment alone (Fig. 4A). These findings indicate that c-Myc and c-Jun are AKT1-dependent regulators.

The study also examined whether c-Myc and c-Jun were regulated in an eIF4E-dependent manner. A375 cells were transfected with eIF4E siRNA for 16 h, followed by treatment with 100 nM RocA for 24 h. The results demonstrated that silencing eIF4E did not affect RocA-induced c-Myc and c-Jun expression (Fig. 4D), compared to RocA treatment alone (Fig. 4A). This evidence suggests that c-Myc and c-Jun are eIF4E-independent regulators.

Previous research demonstrated that eIF4F inhibitor treatment induces cell apoptosis and inhibits cell proliferation through BMF upregulation in melanoma with BRAF and NRAS mutations [14]. In addition, inhibition of the PI3K/AKT pathway or direct pharmacological inhibition of eIF-4E has been shown to increase BMF expression [27]. To investigate whether BMF is regulated by AKT1, A375 cells were treated with 100 nM RocA for 0–48 h. The results showed that BMF expression increased at 3 h, peaked at 12 h, and subsequently returned to basal levels (Fig. 4A).

When A375 cells were exposed to 2.5 μM MK-2206 for 2 h followed by 100 nM RocA treatment for 0–48 h, BMF expression showed a gradual increase over time (Fig. 4B). However, the combination of RocA and U0126 treatment showed no significant effect on BMF expression compared to RocA treatment alone (Fig. 4C). These findings suggest that BMF regulation is AKT1-dependent but not ERK1/2-dependent.

To further examine the apoptotic response induced by RocA, additional pro-apoptotic BH3-only proteins, including PUMA, Bax, and Bad, were examined. Among these, only PUMA showed slight upregulation after 100 nM RocA treatment for 0–48 h (Fig. 4A). Neither U0126 (Fig. 4C) nor MK-2206 (Fig. 4B) treatment affected PUMA expression.

The administration of RocA did not alter Bax and Bad protein levels (Fig. 4A). However, combined MK-2206 and RocA treatment resulted in minimal upregulation of Bax expression (Fig. 4B), while combined U0126 and RocA treatment showed moderate upregulation of Bax expression (Fig. 4C).

### 3.5 RocA Treatment Activates AKT1-Dependent eIF4E

**Figure 5:**
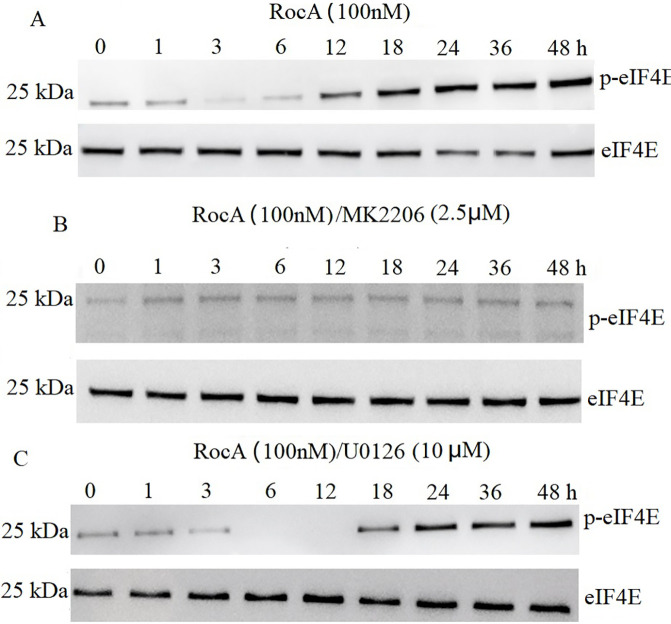
eIF4E is AKT1-dependent following RocA treatment. (**A**) A375 cells were treated with 100 nM RocA for 48 h, and the levels of p-eIF4E and total eIF4E were detected by western blotting. (**B**) A375 cells were co-treated with 100 nM RocA and 2.5 μM MK-2206 for 48 h, and p-eIF4E/eIF4E expression levels were analyzed by western blotting. (**C**) A375 cells were co-treated with 100 nM RocA and U0126 (10 µM) for 48 h, and p-eIF4E/eIF4E levels were analyzed by western blotting

Akt functions as an apoptotic regulator that, when activated, contributes to clinical drug resistance by inhibiting apoptosis and activating eIF4E [20]. Preliminary experiments demonstrated that exposure of A375 cells to 1.25–5 μM MK-2206 for 4 h resulted in complete inhibition of p-AKT1 without causing significant cytotoxicity. Therefore, 2.5 μM MK-2206 was selected as the optimal concentration for subsequent studies. The present investigation revealed increased p-eIF4E expression following RocA treatment (Fig. 5A). MK-2206 treatment suppressed both RocA-induced AKT1 reactivation (Fig. 4B) and p-eIF4E expression (Fig. 5B). In contrast, treatment with the ERK1/2 inhibitor U0126 (10 μM) (Fig. 5C) did not affect RocA-induced p-eIF4E expression compared to RocA treatment alone. These findings suggest that RocA-induced p-eIF4E expression occurs in an AKT1-dependent but ERK1/2-independent manner.

### 3.6 AKT1 Inhibition Enhanced RocA-Induced Cell Apoptosis

**Figure 6:**
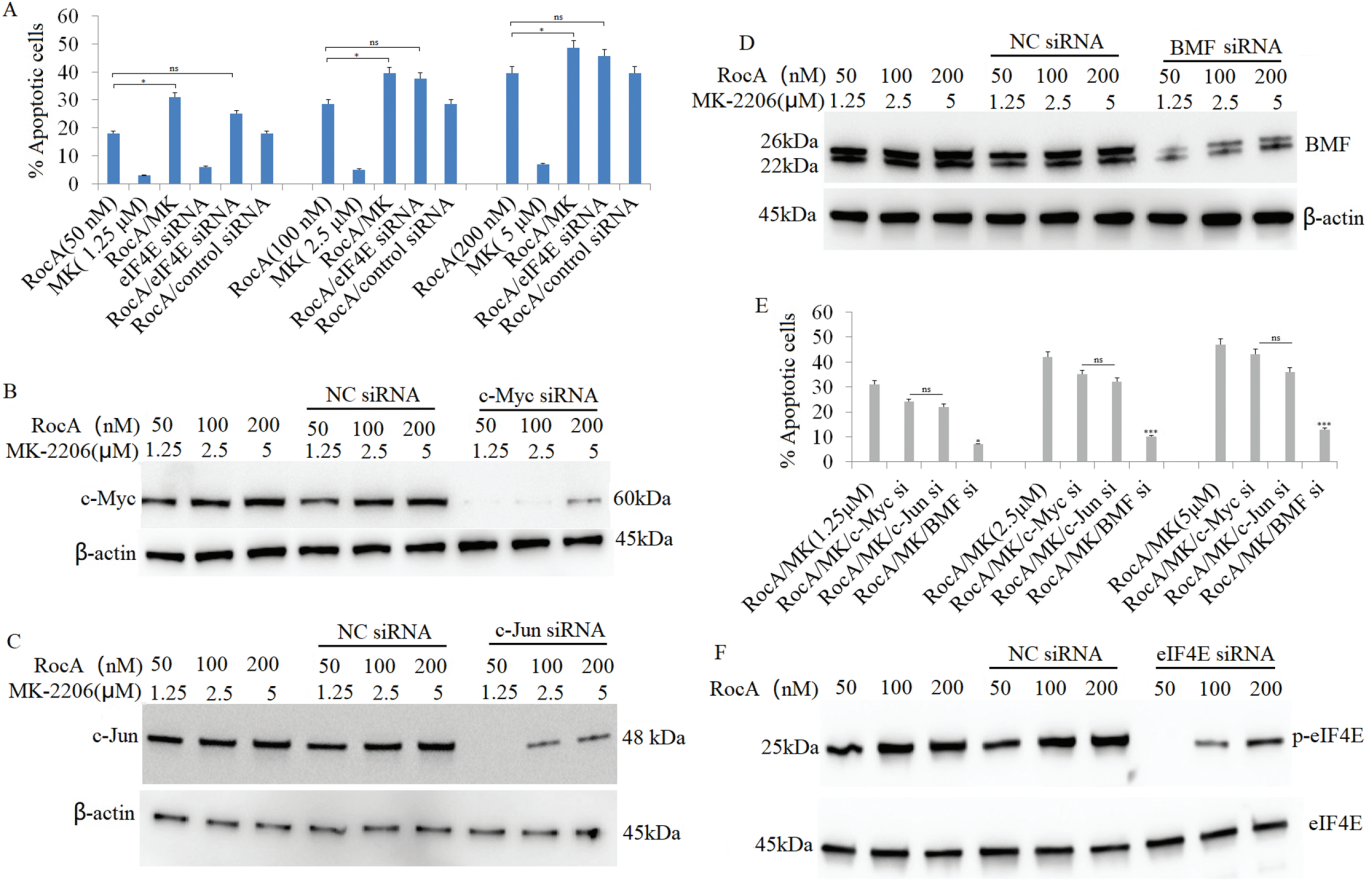
AKT1 inhibition augments RocA-induced apoptosis by inducing BMF expression. (**A**) A375 cells were treated with RocA (50, 100, and 200 nM) and MK-2206 (1.25, 2.5, and 5 μM) for 48 h, or transfected with eIF4E siRNA for 16 h, followed by treatment with RocA (50, 100, and 200 nM) for 48 h. Cell apoptosis was evaluated using the TUNEL assay. ns, not significant, *p < 0.05. (**B**–**D**). A375 cells were transfected with c-Myc siRNA, c-Jun siRNA, or BMF siRNA for 16 h, followed by co-treatment with RocA (50, 100, and 200 nM) and/or MK-2206 (1.25, 2.5, and 5 μM) for 48 h. Protein levels of c-Myc (**B**), c-Jun (**C**), and BMF (**D**) were analyzed by western blotting. (**E**) Protein band intensities were quantified using Image J software, and the relative expression ratios of c-MYC/β-actin, c-BMF/β-actin, and c-Fos/β-actin were calculated. Data are presented as the mean ± SEM of two independent experiments. ns, not significant, *p < 0.05, ***p < 0.001. (**F**) A375 cells were transfected with EIF4E siRNA for 16 h and then co-treated with RocA (50, 100, and 200 nM) for 48 h. eIF4E expression levels were analyzed by western blotting

RocA treatment effectively inhibited cell proliferation and promoted apoptosis in A375 cells, but failed to induce apoptosis in A375R cells. Previous research indicates that the antiproliferative efficacy of the AKT inhibitor MK-2206 alone is limited in hepatocellular carcinoma cells [28], and it does not affect *in vitro* and *in vivo* A375 cell growth or survival. However, while single-agent MK-2206 inhibited phospho-AKT signaling, targeting AKT1 using siRNA for 48 h induced apoptosis in melanoma cells [29]. Based on these findings, the current study investigated whether combined AKT1 inhibition using MK-2206 enhances the antitumor efficacy of RocA by promoting apoptosis in A375 cells. Cells were co-treated with MK-2206 (1.25, 2.5, and 5 μM) and RocA (50, 100, and 200 nM) for 48 h. While MK-2206 alone did not induce apoptosis, the combination treatment significantly induced apoptosis compared to RocA treatment alone (Fig. 6A and Fig. S5).

Since c-Myc, c-Jun, and BMF were regulated by AKT1, this study examined whether these proteins contributed to AKT1-knockdown-induced apoptosis. A375 cells were transfected with c-Myc siRNA, c-Jun siRNA, or BMF siRNA for 16 h, followed by co-treatment with RocA (50, 100, and 200 nM) and MK-2206 (1.25, 2.5, and 5 μM) for 48 h. Western blotting confirmed that siRNA transfection effectively inhibited the expression of their respective proteins (Fig. 6B–D), resulting in decreased apoptosis compared to cells receiving combined RocA and MK-2206 co-treatment (Fig. 6A and Fig. S5).

BMF knockdown significantly inhibited apoptosis induced by combined RocA and MK-2206 treatment (Fig. 6E). In contrast, silencing c-Myc or c-Jun only partially inhibited apoptosis, and these effects did not reach statistical significance (Fig. 6E).

Given that eIF4E expression is regulated by AKT1 and that AKT1 inhibition enhanced RocA-induced apoptosis, this study examined whether targeting eIF4E could similarly increase RocA-induced apoptosis. A375 cells were transfected with eIF4E siRNA for 16 h, followed by RocA treatment (50, 100, and 200 nM) for 48 h. eIF4E knockdown effectively inhibited RocA-induced eIF4E expression (Fig. 6F). While eIF4E siRNA modestly increased RocA-induced apoptosis, the difference was not statistically significant (Fig. 6A). These findings suggest that AKT1 inhibition enhances RocA-induced cell apoptosis primarily through BMF upregulation.

### 3.7 RocA Treatment Inhibits Bcl-2 and Mcl-1 Expression

**Figure 7:**
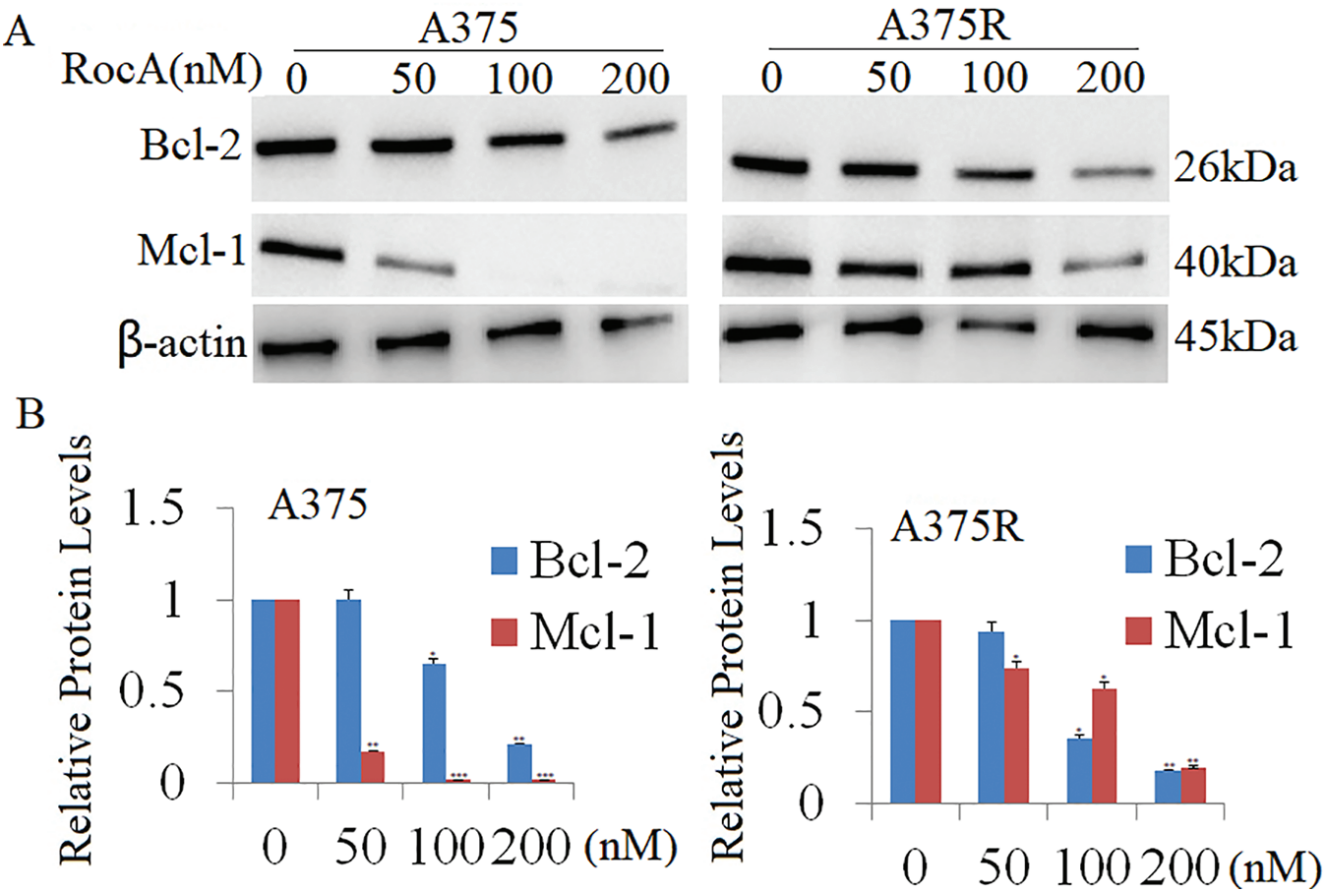
Effect of RocA treatment on Bcl2 and Mcl-1 expression. (**A**) A375 and A375R cells were treated with RocA (50, 100, and 200 nM) for 48 h, and the expression levels of Bcl-2 and Mcl-1 were detected by western blotting. The membranes were reprobed with β-actin antibody to verify equal protein loading. (**B**) Results represent the mean ± SEM from two independent experiments. *p < 0.05, **p < 0.01, ***p < 0.001

Previous research has shown that silencing eIF4A or eIF4E reduces melanoma cell proliferation and invasion [9]. Additionally, eIF4E has been demonstrated to promote neutrophil survival via regulation of the anti-apoptotic proteins Bcl-2 and Mcl-1 [30]. In the current study, A375 and A375R cells were treated with RocA (50, 100, and 200 nM) for 48 h, resulting in significantly decreased Bcl-2 and Mcl-1 expression in both cell lines (Fig. 7A,B). These findings suggest that RocA-induced inhibition of cell viability may be mediated, at least in part, by the downregulation of Bcl-2 and Mcl-1. Further investigation is required to elucidate this mechanism and examine the direct or indirect regulation of Bcl-2 and Mcl-1 expression by AKT1.

### 3.8 Pharmacological Inhibition of eIF4F, AKT1, and EZH2 Enhances the Antitumor Effect In Vitro

**Figure 8:**
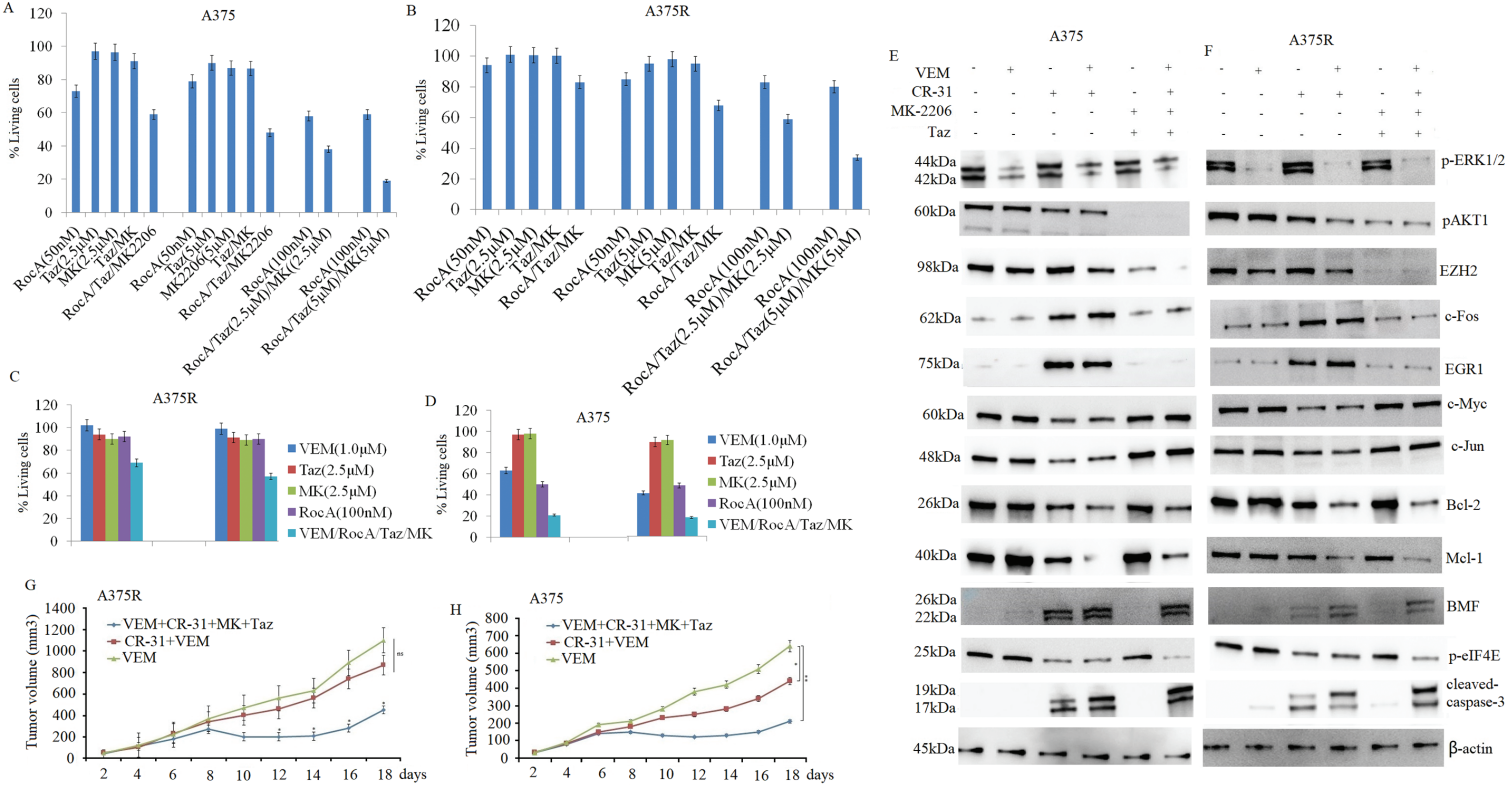
Combined inhibition of eIF4F, AKT1, and EZH2 enhances sensitivity to VEM *in vitro* and *in vivo*. A375 (**A**) and A375R (**B**) cells were treated with RocA (50 and 100 nM), MK-2206 (2.5 and 5 μM), and tazemetostat (2.5 and 5 μM) for 72 h. Cell proliferation was detected by the MTT assay. A375R (**C**) and A375 (**D**) cells were treated with RocA (100 nM), tazemetostat (2.5 and 5 μM), MK-2206 (2.5 and 5 μM), and VEM (1 and 10 μM) for 72 h, and cell proliferation was again detected by the MTT assay. For the short-term therapeutic response group, agents were administered once a day for 5 consecutive days. Tumor tissues were collected and analyzed by western blotting for protein expression in A375 (**E**) and A375R (**F**) tumor tissues. For the *in vivo* xenograft study, A375R (**G**) or A375 (**H**) cells (5 × 10^6^) in 200-µL suspension were subcutaneously injected into mice. After 5 days, the mice were randomized into five treatment groups and treated for 14 days, as described in the Materials and Methods. Tumor volume was measured and plotted over time. Data are presented as mean ± SEM. ns, not significant; *p < 0.05; **p < 0.01

Previous research [31] demonstrated that combined VEM and RocA treatment exhibited modest effects on cell apoptosis and proliferation inhibition in A375R cells, though these effects were temporary owing to acquired resistance [9]. Consistent with this, our earlier findings indicated that combined inhibition of eIF4F with either EZH2i or AKT1i enhanced the sensitivity of A375 cells to eIF4F inhibition. To investigate whether pharmacological inhibition of eIF4F, AKT1, and EZH2 enhances the antitumor effect *in vitro*, A375 cells were co-treated with RocA (50 and 100 nM), tazemetostat (2.5 and 5 μM), and MK-2206 (2.5 and 5 μM) for 72 h. In line with previous studies indicating that AKT1 and EZH2 inhibitors alone have minimal impact on cell growth or apoptosis [9], the current study revealed that the triple-drug combination of MK-2206 + tazemetostat + RocA significantly enhanced the antitumor effect in A375 cells, as measured by the MTT assay (Fig. 8A). Similar effects were observed in A375R cells *in vitro* (Fig. 8B). Further investigation examined whether combined RocA, tazemetostat, and MK-2206 treatment enhanced A375R cell sensitivity to VEM. A375R cells were co-treated with VEM (1 and 10 μM), tazemetostat (2.5 μM), and MK-2206 (2.5 μM) for 72 h. This triple-drug combination significantly improved A375 cell sensitivity to VEM (Fig. 8C) and enhanced VEM’s antitumor efficacy in VEM-sensitive A375 cells (Fig. 8D).

### 3.9 Combined Pharmacological Inhibition of eIF4F, AKT1, and EZH2 Overcomes VEM Resistance In Vivo

Having established that combined inhibition of eIF4A, AKT1, and EZH2 effectively overcomes VEM resistance *in vitro*, we proceeded to investigate the therapeutic potential of this combination *in vivo* and its effect on VEM sensitivity.

For the *in vivo* study, CR-31, a synthetic rocaglate with superior pharmacokinetic properties compared to RocA, was used. A375R cells (5 × 10^6^) were subcutaneously injected into female BALB/c-nu mice (4–6 weeks old, weighing 16–18 g). After 5 days of tumor establishment, mice were randomized into three treatment groups, as previously described. Tumor volumes were measured every 2 days for 19 days. The VEM monotherapy group exhibited rapid tumor growth, while both the combination therapy groups (VEM + CR-31 + MK-2206 + tazemetostat and CR-31 + VEM) demonstrated significant tumor growth reduction compared to the VEM-only group (Fig. 8E).

In A375 xenograft models, the combination treatments exhibited markedly enhanced antitumor efficacy compared to VEM monotherapy. The (VEM + CR-31 + MK-2206 + tazemetostat) group and the (CR-31 + VEM) group demonstrated tumor growth inhibition rates of 81.8% and 60%, respectively, which were substantially higher than the rate of 13.6% observed in the VEM-only group (Fig. 8F). Both combination regimens demonstrated significantly superior efficacy compared to VEM monotherapy. Importantly, no notable toxicity or adverse effects were observed in any treatment groups when compared with the VEM-only group. Furthermore, CR-31 alone demonstrated antitumor activity in VEM-sensitive A375 cells, while the triple combination (CR-31 + MK-2206 + tazemetostat) showed a significantly enhanced sensitivity to VEM treatment compared to the CR-31 + VEM combination.

Subsequently, we examined the molecular mechanisms underlying the effects of CR-31 and the combined treatment on both VEM-sensitive and VEM-resistant A375 xenograft models. After 5 days of tumor establishment, mice in the short-term therapeutic response group were randomized into six treatment groups and treated as previously described for 5 days. Xenograft tumors were harvested 6 h after the final treatment, and protein expression was analyzed by western blotting. The four-drug combination therapy (VEM + CR-31 + MK-2206 + tazemetostat) resulted in a significant reduction in p-AKT1, EZH2, and eIF4E expression compared to other treatment groups (Fig. 8E,F). Additionally, the combination therapy significantly upregulated pro-apoptotic proteins and downregulated growth-promoting proteins in both A375 (Fig. 8G) and A375R (Fig. 8H) tumor tissues. These findings align with the *in vitro* results.

## 4 Discussion

Previous research has demonstrated that eIF4F complex activity contributes to drug resistance in melanoma cells [9,32]. While inhibition of the eIF4F complex increases melanoma cell sensitivity to VEM [9], this inhibition also leads to ERK1/2 signal hyperactivation [8], which contributes to VEM resistance in melanoma. Currently, there is limited systematic research examining the relationship between eIF4F complex inhibition and hyperactivated ERK1/2 signaling.

This investigation revealed that eIF4F complex inhibition through RocA treatment suppressed cell proliferation in both VEM-sensitive A375 and VEM-resistant A375R cells, although cell apoptosis was induced exclusively in A375 cells. Additionally, A375 cells demonstrated greater sensitivity to RocA. Notably, the combination of VEM and RocA therapy enhanced VEM sensitivity in both cell types.

Recent research by Valcikova et al. [8] demonstrated that RocA treatment induces ERK1/2 hyperactivation after 20 h of exposure. In the present study, we continuously monitored p-ERK1/2 expression in A375 cells during RocA treatment from 1 to 48 h. The results showed early ERK1/2 activation, with a gradual increase peaking at 12 h, followed by a gradual decrease to baseline levels after 48 h. While RocA treatment affects cell proliferation and apoptosis through the regulation of c-Myc, c-Jun, EGR1, and c-Fos protein expression [8], the regulatory mechanisms and functional pathways remain to be elucidated.

EZH2 serves a crucial function in melanoma progression [33] and mediates treatment resistance [34]. Research indicates that activated ERK1/2 stimulates EZH2 signals [13,18], establishing EZH2 as a critical therapeutic target in cancer. However, the mechanisms underlying elevated EZH2 levels in melanomas remain unclear. Cha et al. [19] demonstrated that Akt-mediated phosphorylation of EZH2 can enhance its activity, potentially contributing to oncogenesis. In the present study, our findings indicate that RocA treatment reactivates EZH2 through an ERK1/2-dependent pathway, independent of AKT1 signals. The sustained elevation of EZH2 levels despite p-ERK1/2 returning to baseline after 48 h may be attributed to both the extended half-life of EZH2 protein and potential late-stage RocA-induced proteins regulating EZH2. Further investigation is required to elucidate these specific mechanisms. Subsequent experiments demonstrated that combining RocA with EZH2 inhibitors or EZH2 siRNA knockdown produced significant antitumor effects, suggesting that EZH2 reactivation contributes to RocA resistance.

Elevated EGR1 and c-Fos expression has been shown to correlate with cell proliferation. In the present study, RocA treatment elevated EGR1 and c-Fos expression in a time-dependent manner, reaching peak levels at 48 h. Consistent with the findings of Valcikova et al., who demonstrated that c-Fos and EGR1 protein levels positively correlated with increased ERK activity [8], our results further demonstrate that c-Fos and EGR1 positively correlated with increased EZH2 expression. To determine whether EZH2 inhibition could enhance the anticancer effects of RocA through modulation of c-Fos and EGR1, A375 cells were transfected with EGR1 or c-Fos siRNA and subsequently treated with RocA. The results indicated that silencing c-Fos or EGR1 via siRNA significantly reduced RocA-induced cell proliferation. These findings suggest that elevated c-Fos and EGR1 expression, regulated by ERK/EZH2 signals, may contribute to RocA resistance. While RocA treatment induced apoptosis specifically in A375 cells, targeting ERK1/2 or EZH2 showed no significant effect on apoptosis, indicating potential involvement of alternative signaling pathways in apoptosis and cell proliferation following RocA treatment.

Akt, a key regulator of apoptosis, is frequently activated in numerous cancers and may contribute to drug resistance *in vitro* and *in vivo* [19]. Akt prevents apoptosis and contributes to clinical drug resistance through eIF4E activity [20]. Recent research demonstrated that RocA treatment downregulated ERK1/2-independent c-Myc and c-Jun expression [8]. The current study revealed that RocA treatment reactivated AKT1, which in turn led to AKT1-dependent downregulation of c-Myc and c-Jun expression and AKT1-dependent upregulation of eIF4E activity. Following AKT1 inhibition, RocA-treated cells exhibited a marked increase in c-Myc and c-Jun expression along with a significant reduction in eIF4E phosphorylation. In contrast, ERK1/2 inhibition did not affect c-Myc and c-Jun expression and eIF4E activity, confirming that these effects occur via an ERK-independent but AKT1-dependent mechanism. Furthermore, the combination of AKT1 inhibition and RocA treatment induced significant cell apoptosis, whereas silencing c-Myc, c-Jun, or eIF4E individually reduced cell apoptosis to varying degrees under the same conditions. These results indicate that AKT1 partially controls cell apoptosis through the regulation of c-Myc, c-Jun, and eIF4E.

Boussemart et al. [9] reported that inhibition of the eIF4F complex promotes apoptosis by increasing pro-apoptotic BMF, degrading eIF4G, and reducing melanoma cell proliferation. This study examined the effects of RocA treatment on BMF and other pro-apoptotic BH3-only proteins, including PUMA, Noxa, Bim, Bax, and Bad. The results showed RocA treatment initially upregulated BMF expression, followed by a significant decrease in BMF expression after AKT1 reactivation. Inhibition of AKT1 restored BMF expression, while inhibition of ERK1/2 or EZH2 had no effect, indicating that BMF regulation is AKT1-dependent. However, in triple-negative breast cancer (TNBC), EZH2i/AKTi potentially eliminates TNBC cells through a shared BMF activation pathway [35], suggesting that BMF regulation may be tissue-specific. In addition, RocA treatment demonstrated no effect on Noxa, Bim, and Bax protein levels, while slightly elevating PUMA and Bad expression, indicating limited involvement of other pro-apoptotic BH3-only proteins in cell growth and apoptosis following RocA treatment in A375 melanoma cells.

BMF knockdown significantly reduced RocA-induced A375 cell apoptosis and also diminished apoptosis following the combined RocA and AKT1 inhibition treatment. These findings indicate that RocA induces apoptosis primarily through BMF activation, and that AKT1 inhibition enhances RocA-induced apoptosis primarily through BMF activity. Additionally, the anti-apoptotic proteins Bcl-2 and Mcl-1 were found to be overexpressed in A375R cells. RocA treatment significantly reduced Bcl-2 and Mcl-1 expression, and this downregulation occurred independently of ERK1/2 or AKT1 signaling. These findings imply that Bcl-2 and Mcl-1 contribute to RocA resistance in melanoma cells. Additional research is necessary to elucidate the precise role of the eIF4F complex in regulating Bcl-2 and Mcl-1 expression.

Previous research has demonstrated that AKT or EZH2 inhibitors alone do not effectively eliminate TNBCs but demonstrate efficacy when combined in a dose-dependent manner [21]. The present study shows that AKT or EZH2 inhibitors, either alone or in combination, enhance the sensitivity of A375 and VEM-resistant A375R melanoma cells to RocA treatment. RocA, AKT, and EZH2 inhibitors demonstrated synergistic effects and collectively promoted tumor regression in A375 and A375R models *in vivo*. Mechanistically, RocA treatment was found to reactivate AKT or ERK1/2-EZH2 signals, inhibit pro-apoptotic signals, and activate proliferative signals, leading to RocA resistance. The addition of EZH2 and AKT inhibitors enhanced cell sensitivity to RocA primarily through the inhibition of the AKT1 pathway, which triggers the expression of pro-apoptotic proteins such as BMF, c-Myc, c-Jun, and PUMA, while concurrently inhibiting the anti-apoptotic factor eIF4E. Furthermore, these inhibitors suppressed the ERK1/2-EZH2 axis, thereby blocking the expression of Bcl-2, Mcl-1, c-Fos, and EGR1. However, the precise mechanisms by which the eIF4F complex regulates EZH2 and/or AKT function in this context remain unclear.

Combined pharmacological inhibition of eIF4A, AKT1, and EZH2 yielded the most pronounced inhibition of cell proliferation and robust inhibition of apoptosis in both VEM-sensitive (A375) and VEM-resistant (A375R) cells. Combined pharmacological inhibition of eIF4A and VEM demonstrated a significant antitumor effect in sensitive A375 cells and a moderate antitumor effect in resistant A375 cells *in vitro* and *in vivo*. Moreover, the combined inhibition of eIF4E, AKT1 (MK-2206), and EZH2 (tazemetostat), together with VEM, exhibited markedly stronger antitumor activity compared to the combination of the eIF4A inhibitor and VEM. These findings indicate that simultaneous targeting of eIF4E, AKT1, and EZH2 inhibitors may effectively overcome VEM resistance, offering a promising therapeutic strategy for melanoma treatment.

This study has several limitations. First, the study utilized only one melanoma cell line, A375, and its VEM-resistant derivative, A375R, necessitating validation with additional cell lines. Second, while ERK1/2, AKT1, and EZH2 regulate the expression of various proteins affecting cell function, it remains unclear whether this protein expression is regulated through pre-transcriptional or post-transcriptional mechanisms, requiring more comprehensive experimental studies. Third, further research is needed to determine how Bcl-2 and Mcl-1 are regulated and whether eIF4F inhibition affects other cellular processes beyond proliferation and apoptosis. Fourth, further investigation is needed to determine whether ERK/AKT signals rewire transcriptional networks or affect protein stability. In conclusion, this study reveals a critical role of eIF4F in the negative regulation of the ERK1/2 pathway in melanoma, consistent with recent research findings [8]. Our findings demonstrate that eIF4F inhibition hyperactivates the ERK1/2-EZH2 signaling pathway, activating c-Fos and EGF1 and leading to eIF4F inhibitor resistance. The findings show that inhibition of the eIF4F complex reactivated AKT1-dependent p-eIF4E and reduced the expression of AKT1-dependent pro-apoptotic proteins BMF, c-Myc, and c-Jun, resulting in decreased cell apoptosis following eIF4F inhibitor treatment. Additionally, inhibition of the eIF4F complex inhibited cell proliferation and induced cell apoptosis by inducing BMF expression and decreasing Bcl-2 and Mcl-1 expression. The results indicate that combining eIF4F complex inhibitors with AKT1 and EZH2 inhibitors enhances the antitumor effect in A375 cells. The triple combination effectively reversed VEM resistance in both VEM-resistant and VEM-sensitive melanoma cells. These research findings advance the understanding of the molecular mechanisms underlying eIF4F complex inhibitor therapy in melanoma and shed light on how melanoma develops resistance to targeted therapy. Importantly, this study provides novel insights into potential strategies for improving treatment outcomes for patients with BRAFmutant melanoma.

## Supplementary Materials













## Data Availability

All data generated or analyzed during this study are included in this published article.

## Supplementary Materials

The supplementary material is available online at https://www.techscience.com/doi/10.32604/or.2025.071034/s1.
