# Supplementary figures and images for: Combinational Inhibition of the eIF4F Complex, AKT1, and EZH2 Enhances Anticancer Effects in BRAFV600E Mutant A375 Melanoma Cells
Source: Oncol Res. 2026 Feb 24;34(3):18. doi: 10.32604/or.2025.071034 (PMC12963650; doi:10.32604/or.2025.071034)

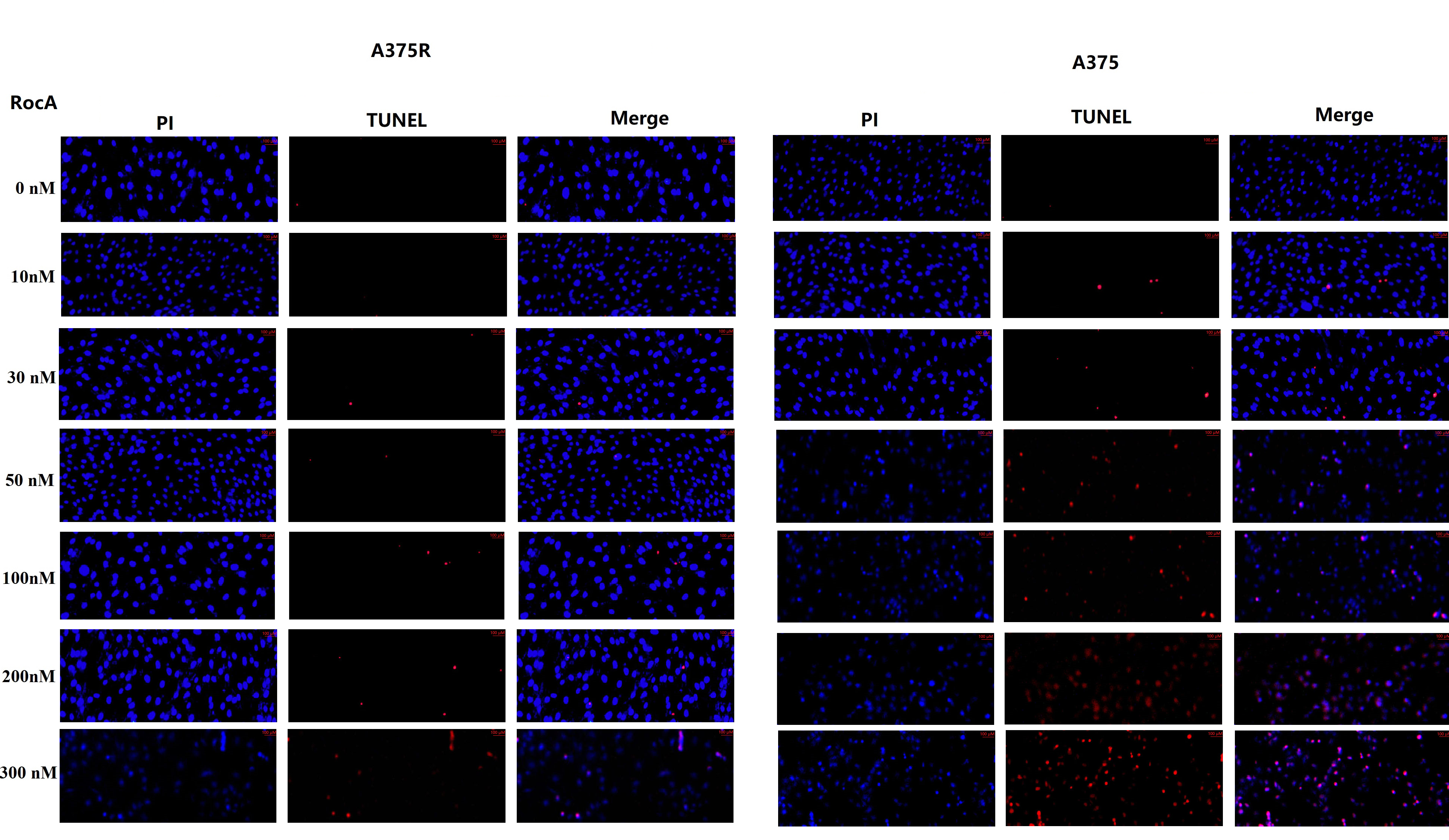

Supplement: Supplementary file 1 [file OncolRes-34-71034-s001.tif]

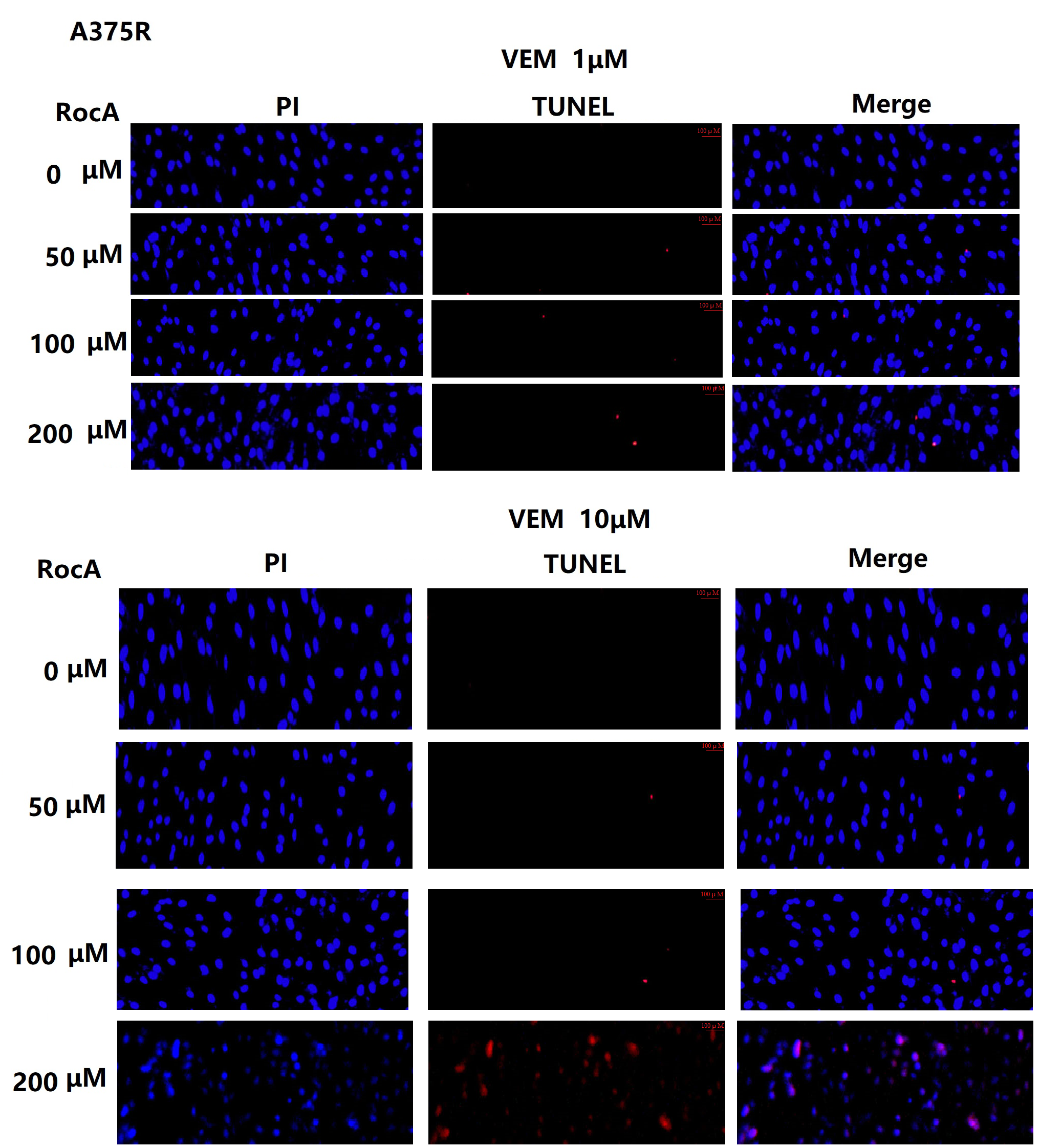

Supplement: Supplementary file 2 [file OncolRes-34-71034-s002.tif]

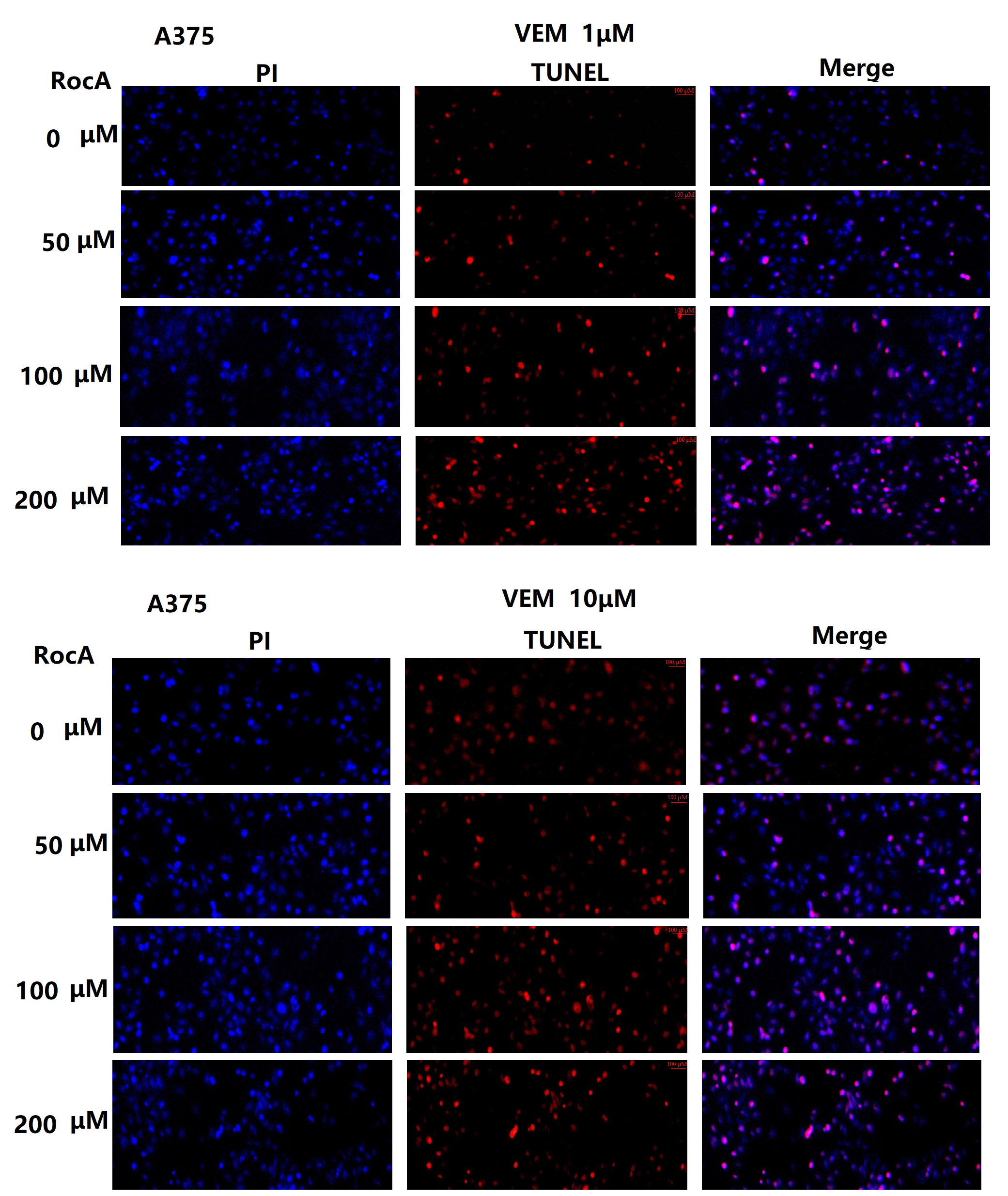

Supplement: Supplementary file 3 [file OncolRes-34-71034-s003.tif]

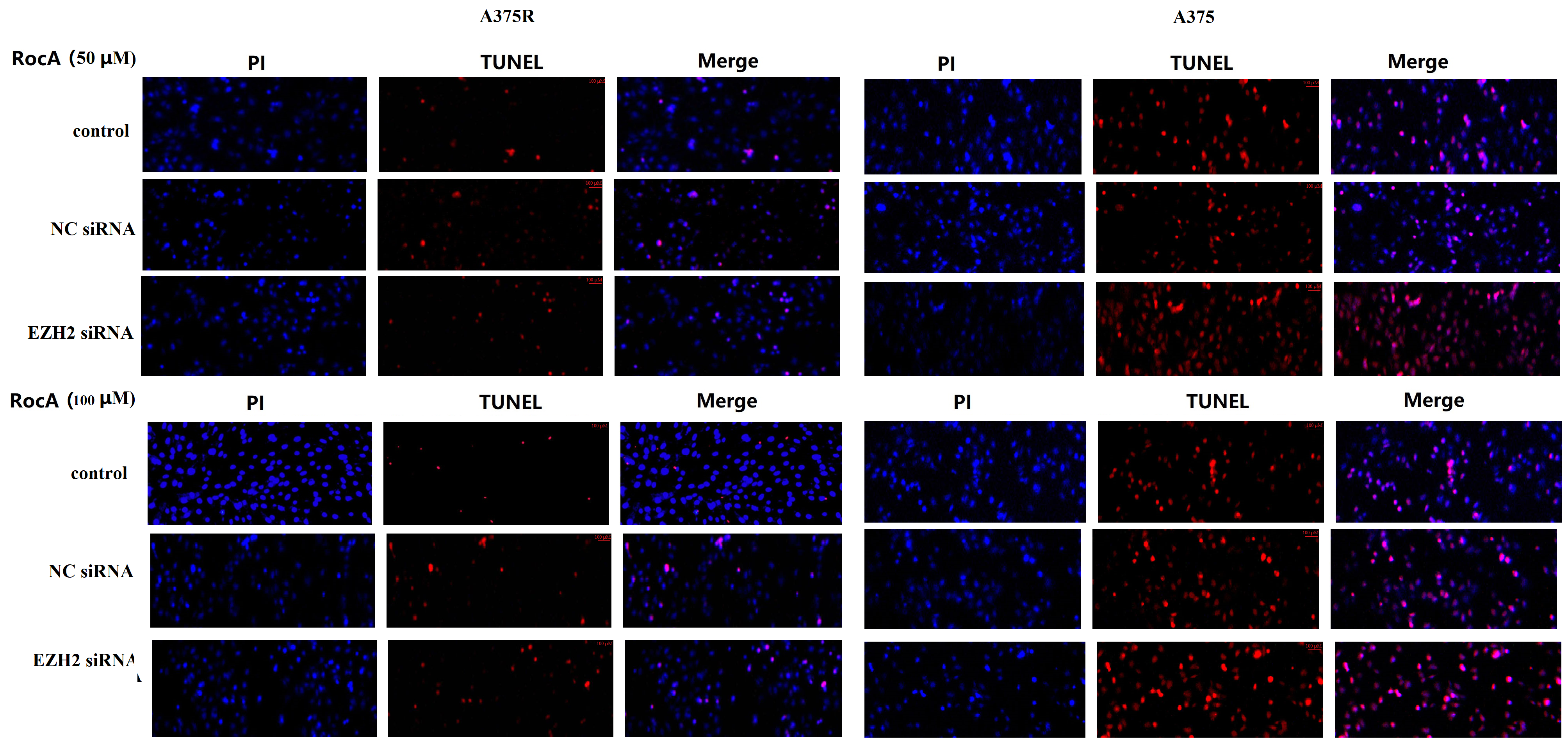

Supplement: Supplementary file 4 [file OncolRes-34-71034-s004.tif]

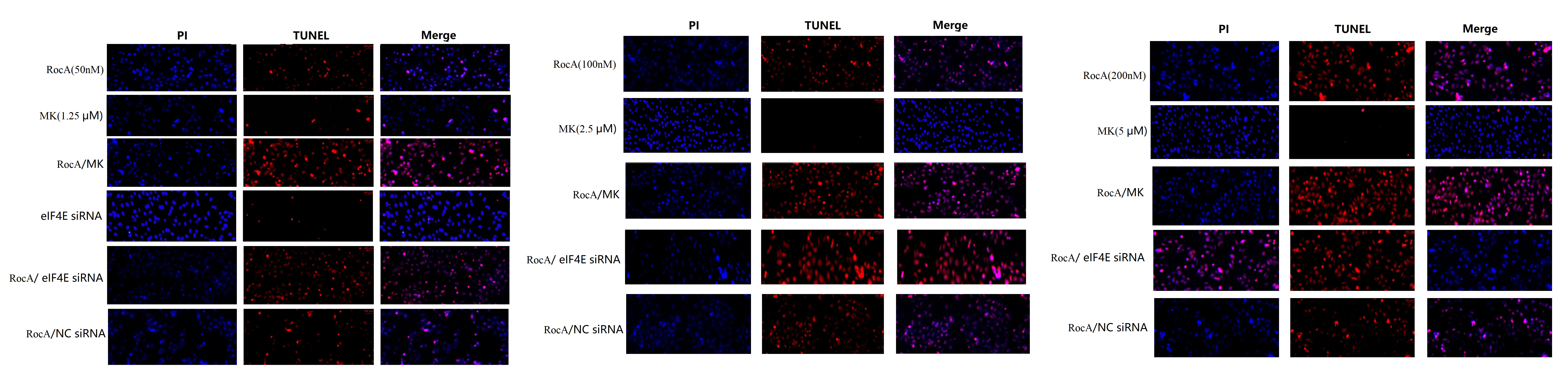

Supplement: Supplementary file 5 [file OncolRes-34-71034-s005.tif]
