# Supplementary material for: Combinational Inhibition of the eIF4F Complex, AKT1, and EZH2 Enhances Anticancer Effects in BRAFV600E Mutant A375 Melanoma Cells
Source: Oncol Res. 2026 Feb 24;34(3):18. doi: 10.32604/or.2025.071034 (PMC12963650; doi:10.32604/or.2025.071034)
Supplement: Supplementary file 6 [file OncolRes-34-71034-s006.docx]

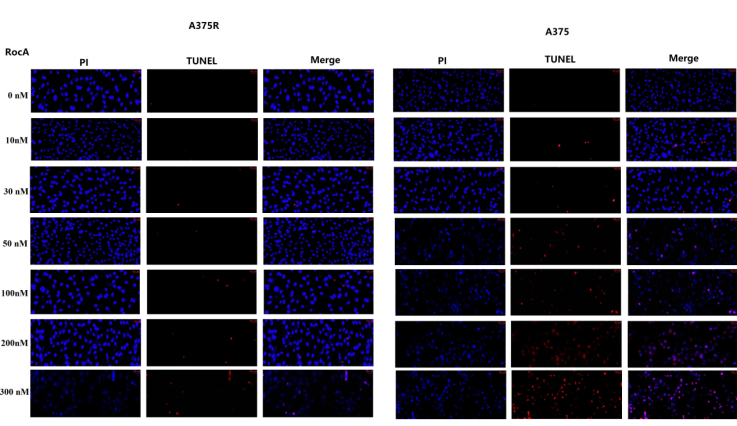


**Figure S1:** A375 and A375R cells were treated with different concentrations of RocA ( 10, 30, 50, 100, 200, and 300 nM ) for 48 hours. Cell apoptosis was detected by TUNEL assay.


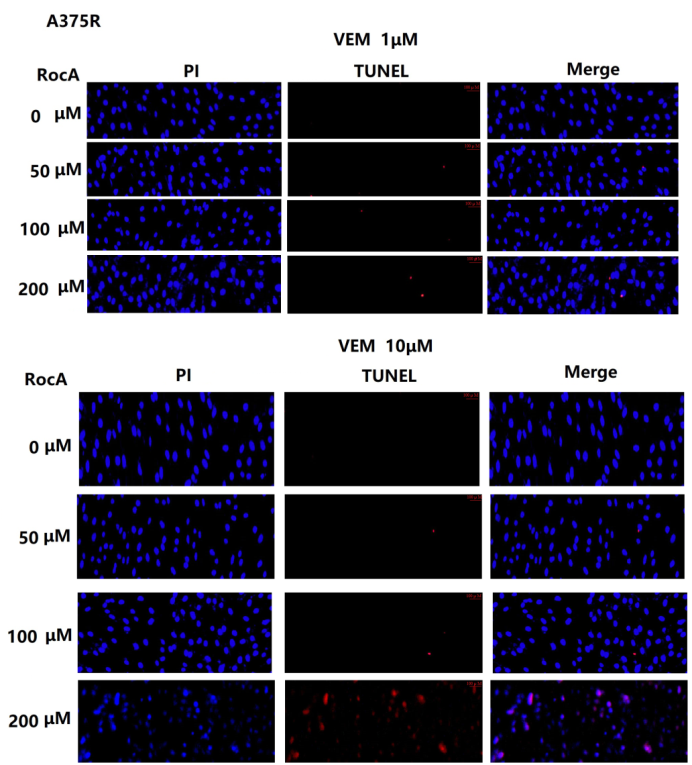


**Figure S2:** A375R cells were co-treated with VEM (1 and 10 μM) and RocA (50, 100, and 200 nM) for 48 hours. Cell apoptosis was detected by TUNEL assay.


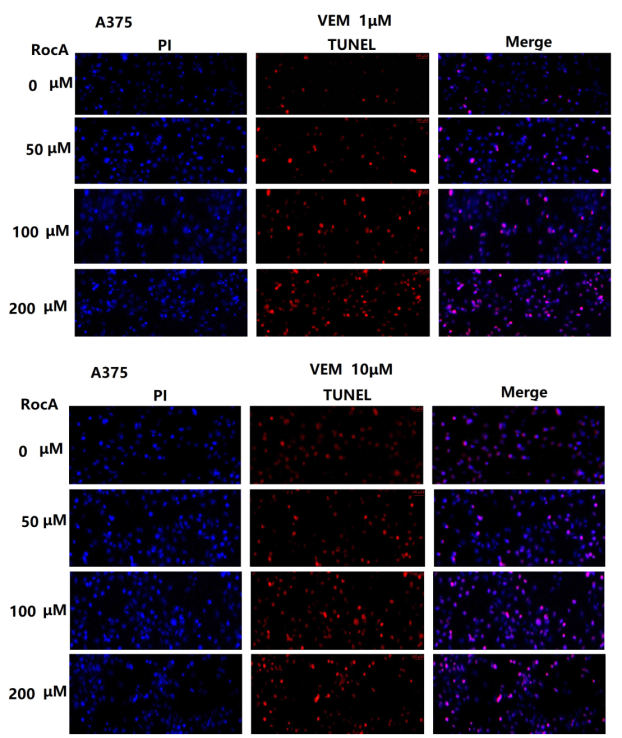


**Figure S3:** A375 cells were co-treated with VEM (1 and 10 μM) and RocA (50, 100, and 200 nM) for 48 hours. Cell apoptosis was detected by TUNEL assay.


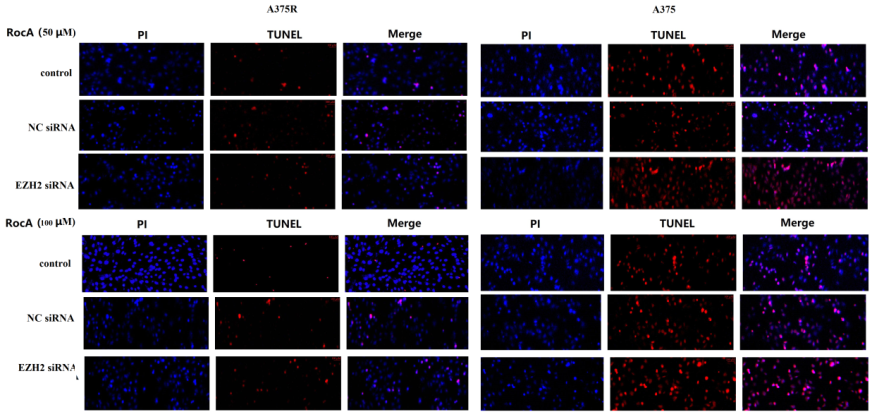


**Figure S4:** A375 cells were transfected with EZH2 siRNA or NC siRNA for 16 hours, followed by RocA treatment (50 and 100 nM) for 48 hours. Cell apoptosis was evaluated using the TUNEL assay.


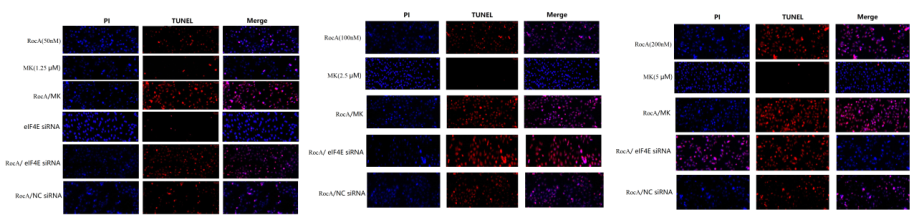


**Figure S5:** A375 cells were treated with RocA (50, 100, and 200 nM) and MK-2206 (1.25, 2.5, and 5 μM) for 48 hours,or transfected with eIF4E siRNA for 16 hours, followed by treatment with RocA (50, 100, and 200 nM) for 48 hours. cell Cell apoptosis was evaluated using the TUNEL assay.
